# In search of immune cellular sources of abnormal cytokines in the blood in autism spectrum disorder: A systematic review of case-control studies

**DOI:** 10.3389/fimmu.2022.950275

**Published:** 2022-10-04

**Authors:** Wared Nour-Eldine, Samia M. Ltaief, Nimshitha P. Abdul Manaph, Abeer R. Al-Shammari

**Affiliations:** Neurological Disorders Research Center, Qatar Biomedical Research Institute, Hamad Bin Khalifa University, Qatar Foundation, Doha, Qatar

**Keywords:** immune cells, cytokines, blood, autism, systematic review, case-control

## Abstract

Abnormal cytokine levels in circulating blood have been repeatedly reported in autism; however, the underlying cause remains unclear. This systematic review aimed to investigate cytokine levels in peripheral blood compartments and identify their potential immune cellular sources in subjects with autism through comparison with controls. We conducted an electronic database search (PubMed, Scopus, ProQuest Central, Ovid, SAGE Journals, and Wiley Online Library) from inception (no time limits) to July 9, 2020, and identified 75 relevant articles. Our qualitative data synthesis focused on results consistently described in at least three independent studies, and we reported the results according to the PRISMA protocol. We found that compared with controls, in subjects with autism, cytokines IL-6, IL-17, TNF-α, and IL-1β increased in the plasma and serum. We also identified monocytes, neutrophils, and CD4+ T cells as potential sources of these elevated cytokines in autism. Cytokines IFN-**γ**, TGF-β, RANTES, and IL-8 were increased in the plasma/serum of subjects with autism, and IFN-**γ** was likely produced by CD4+ T cells and natural killer (NK) cells, although conflicting evidence is present for IFN-**γ** and TGF-β. Other cytokines—IL-13, IL-10, IL-5, and IL-4—were found to be unaltered in the plasma/serum and post-stimulated blood immune cells in autistic individuals as compared with controls. The frequencies of T cells, monocytes, B cells, and NK cells were unchanged in subjects with autism as opposed to controls, suggesting that abnormal cytokines were unlikely due to altered cell numbers but might be due to altered functioning of these cells in autism. Our results support existing studies of abnormal cytokines in autism and provide comprehensive evidence of potential cellular sources of these altered cytokines in the context of autism.

**Systematic Review Registration:**

https://www.crd.york.ac.uk/prospero/display_record.php?ID=CRD42020205224, identifier [CRD42020205224].

## Introduction

Autism spectrum disorder (ASD) is a heterogeneous neurodevelopmental disorder characterized by stereotyped behaviors and interests, impaired communication skills, and difficulties in social interactions. Autism is present in early childhood and affects approximately 1 in 100 children (1), according to World Health Organization epidemiological data (2022). Despite the high prevalence of autism, the etiologies and pathogenesis are still not clearly defined but are known to involve genetic and environmental factors and complex interactions between them.

Emerging evidence supports the link between immune disruption and autism. Epidemiological studies indicate that maternal infection during pregnancy is associated with an increased risk of autism development in the offspring (2–4). These correlation studies are also supported by animal models of maternal immune activation; these models give rise to offspring that exhibit behavioral and neuropathological deficits relevant to autism (3). In addition, a family history of autoimmunity and autoantibodies is also associated with an increased risk of autism (5, 6). An increased number of autoantibodies are produced by mothers with a family history of autoimmunity; these autoantibodies may target specific proteins in the fetal brain that affect neurodevelopment, which might lead to autism-related behaviors in the offspring (5, 6). In contrast, neonatal studies have shown that immune dysregulation has been detected at birth in children who later develop autism. For example, several studies have identified a correlation between abnormal levels of several cytokines in neonatal bloodspots, such as IL-1β, IL-6, IL-8, and RANTES, and increased risk of developing autism (7–11). Together, these studies indicate that abnormal immune factors are present during early development, before the onset of autism symptoms, and accordingly suggest that immune dysfunction is more likely to be a contributing cause of autism rather than an outcome (12).

Remarkably, immune dysregulation persists postnatally, and ongoing inflammation has been observed in individuals with autism. Several studies have revealed abnormal cytokine levels in the peripheral blood of subjects with autism, which were also associated with increased severity of autism symptoms (13, 14). Similarly, there is evidence of increased inflammatory cytokines in brain tissues and cerebrospinal fluid, as well as active neuroinflammation in the brains of individuals with autism (13–15). Cytokines play a substantial role in the brain by signaling other cells to remove dead and damaged neurons and conducting physiological and neuroprotective functions. Increased levels of inflammatory cytokines in the peripheral immune system are also correlated with increased expression of these cytokines in brain neurogenic niches, which could lead to disrupted synaptic plasticity and impaired behavioral outcomes in autism (6, 16, 17). Therefore, persistent abnormalities in peripheral cytokine levels in subjects with autism might reflect changes in cytokine levels in the brain, which are correlated with behavioral deficits in autism.

In this review, we focus on studies that investigated immune profiles in the peripheral blood of subjects with autism compared with controls. Various studies have addressed the implications of peripheral cytokines in the pathophysiology of autism, as reported in previous systematic reviews and meta-analyses (18, 19). However, these reviews focused on the peripheral levels of cytokines in autism without considering their potential cells of origin. This is important for targeted therapies for autism. The objective of this systematic review is to provide an update on the dysregulation of cytokine levels in autism and associate these altered cytokines with their cellular immune sources. To our knowledge, this is the first review to integrate altered cytokines and their specific cellular sources in autism.

## Methods

This study was designed, reported, and executed based on the Preferred Requirements for Systematic Reviews and Meta-Analyses (PRISMA) (20). The review protocol was registered in the International Prospective Register of Systematic Reviews (registration number CRD42020205224) (21). The registered protocol was amended twice to correct a minor typing error and update the current status of the review by adding more details to the protocol sections without changing the original protocol. The PRISMA checklist used in this study is presented in **Table S1**.

### Inclusion and exclusion criteria

This review focused on studies of immune cells and cytokines in the blood of subjects with autism compared with their matching controls. The inclusion criteria were the following: (1) original articles; (2) case-control studies; (3) sample type: peripheral blood, plasma, or serum; (4) analysis of immune cell percentages; (5) analysis of cytokine levels in plasma, serum, or bloodspot; (6) analysis of cytokine levels in immune cells at baseline or after *in vitro* stimulation; and (7) analysis of protein or gene expression. The exclusion criteria were as follows: (1) animal work; (2) sample type: cerebrospinal fluid, tissues, brain, or cell lines; (3) cases without controls; (4) subjects under medication; (5) analysis of treatment or intervention; (6) analysis of immune cell receptors, transcription factors, or activation markers; and (7) genetic (DNA) analysis.

### Search strategy

We performed electronic searches of six different databases (PubMed, Scopus, ProQuest Central, Ovid, SAGE Journals, and Wiley Online Library) using consistent terms. We applied a search filter to the English language results and original journal articles. No other limits were applied. The search was conducted from inception to July 9, 2020, which means no time limits were applied at the time of our database search on July 9, 2020. The search terms used in the PubMed database are as follows: (autism OR autistic OR ASD) AND (human OR subject OR child OR participant OR patient OR volunteer) AND (blood OR plasma OR serum OR immune OR peripheral OR circulating) AND ((cytokine OR chemokine OR “growth factor” OR interferon OR “tumor necrosis factor” OR “colony stimulating factor” OR interleukin) OR (lymphocyte OR monocyte OR “B cell” OR “T cell” OR “natural killer” OR “dendritic cell” OR neutrophil OR basophil OR eosinophil OR myeloid)). The search terms for the remaining databases are listed in **Table S2**.

**Figure 1:**
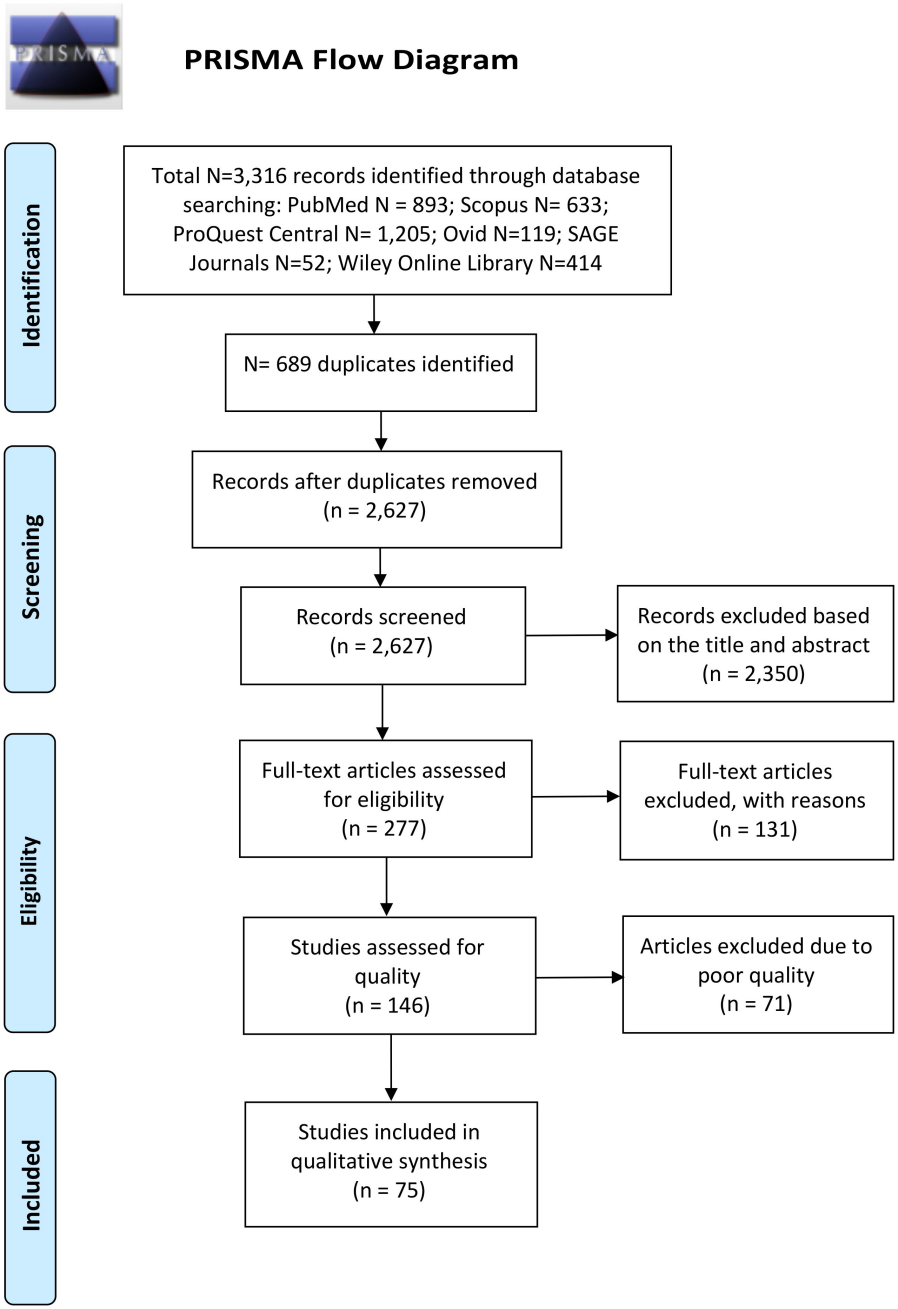
PRISMA flow diagram displaying the different stages of this systematic review and the number of records identified in each stage.

After removing duplicates, we screened the records for relevance based on title and abstract. The full texts of potentially relevant records were retrieved and assessed for eligibility according to our pre-defined inclusion and exclusion criteria, as detailed above. We then assessed the quality and risk of bias of each eligible study using the NIH Quality Assessment Tool of Case-Control Studies (available at: https://www.nhlbi.nih.gov/health-topics/study-quality-assessment-tools), which includes 12 evaluation criteria, and a final rating for each study was given based on the following criteria: 7–12 (good), 4–6 (fair), or 0–3 (poor). Studies with good or fair quality ratings were included in the qualitative synthesis of this review. All steps in the identification, screening, eligibility, and selection of records were performed by two authors independently, followed by discussion and consensus between the two authors, and any disagreement was resolved through discussion with a third author. A PRISMA flow diagram is shown in **Figure 1**.

### Data extraction

We extracted data from the included studies and separated them into two tables in Word documents. These tables list the main outcome (increase, decrease, or no change) in autism compared with controls for either immune cell percentage or cytokine release from immune cells (**Table S3**) and cytokine levels in plasma, serum, or bloodspot (**Table S4**). Other data, such as sample size, age range, sample type, and analysis technique, are also included in **Tables S3** and **S4**. From these two tables, we identified results that were consistently reported in at least three studies and placed these data into three sub-tables (**Tables 1**, **2**, and **3**), which formed the basis of this review. Data were extracted by two authors, and all data were re-checked by at least two independent authors to verify accuracy.

**Table 1:** Baseline levels of cytokines in the blood of individuals with autism compared with controls.

Cytokine affected	Sample type	No. of studies	Age range (in years) (mean ± SD)		Total sample size		% Males	Reported findings in autism compared with controls (in at least N = 3 references)		
			Autism	Controls	Autism	Controls	Autism; Controls	↑	↓	≈
IL-6	Plasma and serum	12	2 to 21 (6.85 ± 2.21)	2 to 21 (7.04 ± 2.08)	540	470	86;75	(22–28)		(29–33)
	Plasma	4	3 to 8 (6.78 ± 2.73)	3 to 8 (6.36 ± 2.43)	134	122	85;73			(29–32)
	Serum	5	2 to 21 (5.91 ± 1.82)	2 to 21 (6.60 ± 1.97)	244	202	86;74	(22–26)		
IL-17	Plasma	5	2.1 to 11.8 (7.11 ± 2.35)	2.6 to 12 (6.83 ± 2.41)	200	168	82;71			(29–31, 34, 35)
	Serum	3	3 to 14.5 (6.70 ± 1.86)	3 to 11 (7.06 ± 1.56)	127	98	89;83	(22, 33, 36)		
TNF-α	Plasma and serum	13	2 to 21 (6.18 ± 2.29)	2 to 21 (7.03 ± 2.17)	615	446	84;70	(24, 29, 33, 37–40		(25, 27, 30–32, 41)
	Plasma	3	3 to 8 (6.67 ± 2.34)	3 to 8 (6.23 ± 1.92)	94	87	89;77			(30–32)
	Serum	5	2 to 21 (5.23 ± 2.11)	2 to 21 (7.33 ± 2.51)	156	142	84;64	(24, 33, 37, 38, 40)		
IL-1β	Plasma and serum	15	2 to 21 (6.43 ± 2.63)	2 to 21 (7.08 ± 2.31)	659	496	85;73	(23–25, 27, 33, 37, 42, 43)		(22, 29, 30, 32, 40, 41, 44)
	Plasma	4	NR (8.08 ± 3.80)	NR (7.43 ± 2.93)	119	102	83;80			(29, 30, 32, 44)
	Serum	6	2 to 21 (5.25 ± 1.95)	2 to 21 (5.76 ± 1.81)	243	202	84;70	(24, 25, 33, 37, 42, 43)		
IFN-γ	Plasma and serum	11	1.58 to 14.5 (6.02 ± 2.21)	2.3 to 12 (7.00 ± 2.08)	564	374	88;73			(22, 25, 27, 29–31, 33, 39–41, 45)
	Plasma	10	2.2 to 16 (6.98 ± 2.51)	2.3 to 16 (6.61 ± 2.30)	474	344	85;77	(32, 46, 47)		(27, 29–31, 39, 41, 45)
	Serum	4	2 to 14.5 (5.17 ± 1.87)	3 to 16 (7.55 ± 1.63)	154	92	93;71			(22, 25, 33, 40)
TGF-β	Plasma and serum	8	2 to 28 (11.46 ± 1.73)	2 to 26 (14.16 ± 2.15)	349	243	89;77	(39, 40, 46, 48)	(35, 49–51)	
	Plasma	6	1.6 to 13 (8.70 ± 1.20)	2 to 14 (9.10 ± 1.70)	300	208	83;73	(39, 46, 48)	(35, 50, 51)	
RANTES	Plasma	3	3 to 8 (4.40 ± 0.83)	2.8 to 8 (4.21 ± 0.98)	167	131	88;59	(31, 52, 53)		
IL-13	Plasma and serum	4	2 to 21 (6.31 ± 2.17)	2 to 21 (5.75 ± 1.99)	196	179	93;79			(24, 27, 30, 31)
IL-12p70	Plasma	4	2.2 to 10 (6.79 ± 2.00)	2.3 to 10 (6.36 ± 1.91)	186	119	87;77			(30, 31, 41, 45)
IL-12p40	Plasma	3	3 to 10 (6.79 ± 2.00)	3 to 10 (6.36 ± 1.91)	169	103	89;75			(30, 31, 41)
IL-10	Plasma and serum	7	2.2 to 10 (6.44 ± 2.32)	2.3 to 12 (6.48 ± 2.11)	225	186	86;74			(25, 29–33, 45)
	Plasma	5	2.2 to 8 (6.78 ± 2.73)	2.3 to 8 (6.36 ± 2.43)	151	138	85;75			(29–32, 45)
IL-8	Plasma and serum	10	1.58 to 10 (5.68 ± 2.03)	2 to 16 (6.97 ± 2.01)	710	397	88;73	(27, 31, 37, 54)		(7, 25, 30, 39–41)
IL-5	Plasma	4	2.2 to 8 (7.10 ± 1.84)	2.3 to 8 (6.86 ± 1.89)	160	133	84;73			(27, 30, 31, 45)
IL-4	Plasma and serum	8	2.2 to 14.5 (6.47 ± 2.46)	2.3 to 11 (6.38 ± 2.11)	330	283	87;78			(22, 27, 29–33, 45)
	Plasma	6	2.2 to 8 (6.78 ± 2.73)	2.3 to 8 (6.36 ± 2.43)	248	225	85;76			(27, 29–32, 45)
IL-2	Plasma and serum	5	2.9 to 10 (7.19 ± 2.32)	2.8 to 10 (7.08 ± 1.93)	277	204	85;81			(27, 30, 32, 33, 41)
	Plasma	4	2.9 to 10 (7.76 ± 2.67)	2.8 to 10 (7.41 ± 2.23)	245	176	84;82			(27, 30, 32, 41)
MCP-1	Plasma	4	1.6 to 6 (5.63 ± 1.77)	3 to 6.6 (5.29 ± 1.69)	1141	1295	88;76			(30, 31, 39, 53)
	Bloodspot	3	Neonates	Neonates	942	1181	84;81			(7, 8, 11)
MIP-1α	Plasma	3	1.58 to 6.17 (8.11 ± 3.65)	2.8 to 6.58 (7.44 ± 3.12)	192	124	89;79			(30, 39, 52)
	Bloodspot	3	Neonates	Neonates	656	962	85;68			(7, 10, 11)
GM-CSF	Plasma	4	2.9 to 10 (6.79 ± 2.00)	2.8 to 10 (6.36 ± 1.91)	266	190	88;77			(27, 30, 31, 41)
Eotaxin	Plasma	4	3 to 10 (6.17 ± 1.74)	3 to 10 (5.86 ± 1.71)	211	138	89;67			(30, 31, 41, 53)

Symbols: ↑, increased; ↓, decreased; ≈, unchanged; in autism compared with controls

GM-CSF, granulocyte monocyte colony-stimulating factor; IFN-γ, interferon-γ; IL, interleukin; MCP-1, monocyte chemoattractant protein-1; MIP-1α, macrophage inflammatory protein; NR, not reported; RANTES, regulated upon activation, normal T cell expressed and presumably secreted; TGF-β, transforming growth factor-β; TNF-α, tumor necrosis factor-α.

**Table 2:** Level of cytokines produced by blood immune cells of individuals with autism, compared with controls, at either baseline or after *in vitro* stimulation conditions.

Cytokine affected	Cell type(s)	Condition (Stimuli^cell type^)	No. of studies	Age range (in years)(mean ± SD)		Total sample size		% Males	Reported findings in autism compared with controls (in at least N = 3 references)		
				Autism	Controls	Autism	Controls	Autism; Controls	↑	↓	≈
IL-6	PBMCs, CD16+ Neutrophils, CD14+ Monocytes	Baseline	6	3 to 15 (6.96 ± 2.56)	4 to 12 (7.76 ± 2.45)	366	335	78;76	(55–60)		
	PBMCs^a^, CD16+ Neutrophils^b^, CD14+ Monocytes^c^	Post-stimulation (PMA/Ionomycin^a^, LPS+ATP^a^, IL-17A^b^, LTA^c^)	4	2.2 to 12 (7.05 ± 2.12)	2.3 to 12 (6.32 ± 2.21)	132	111	86;84	(42, 45, 56, 61)		
IL-17	CD4+ T cells, CD16+ Neutrophils	Baseline	3	NR (6.70 ± 1.80)	NR (6.70 ± 1.76)	120	116	80;32	(56, 58, 62)		
	PBMCs^a^, CD4+ T cells^d^	Post-stimulation (PHA^a^, PMA/Ionomycin^d^, anti-CD3/CD28^d^)	3	2 to 6 (6.65 ± 1.20)	2 to 6 (6.70 ± 1.30)	120	129	86;86	(62–64)		
TNF-α	PBMCs, CD3+, CD8+ or CD4+ T cells	Baseline	3	2.5 to 4.8 (6.90 ± 3.80)	2.2 to 6.1 (8.40 ± 4.00)	252	209	83;76			(57, 59, 65)
	PBMCs^a^, CD14+ Monocytes^c^	Post-stimulation (LPS^a^, PHA^a^, LTA^c^)	3	2.2 to 11.2 (NR)	2.3 to 8.3 (NR)	109	124	82;73	(45, 66, 67)		
IL-1β	PBMCs	Baseline	4	2 to 10 (6.60 ± 2.60)	2 to 17 (7.60 ± 2.70)	277	244	79;76			(57–59, 68)
	PBMCs^a^, CD14+ Monocytes^c^, CD4+ T cells^d^	Post-stimulation (PMA/Ionomycin^a^, LPS+ATP^a^, LPS+BDE-47^a^, LPS^a,c^, LTA^c^, anti-CD3/CD28^d^)	6	2.2 to 11.2 (7.28 ± 2.40)	2.3 to 11 (7.56 ± 2.47)	167	182	77;75	(42, 45, 66, 69–71)		
IFN-γ	PBMCs, CD56+ NK cells	Baseline	3	2.2 to 56 (16.87 ± NR)	2 to 56 (16.70 ± NR)	118	83	84;81	(68, 72, 73)		
	PBMCs^a^, CD4+ T cells^d^, CD56+ NK cells^e^	Post-stimulation (PMA/Ionomycin^a^, tetanus toxoid^a^, PGN^a^, anti-CD3/CD28^a,d^, IL-12+IL-18^e^, K562 cells^e^)	7	2.2 to 56 (10.66 ± 2.04)	2.3 to 56 (10.71 ± 2.15)	294	275	82;78	(61, 69, 70)	(66, 67, 72, 73)	
IL-13	PBMCs	Post-stimulation (PGN, PHA, LPS, tetanus toxoid)	3	2.2 to 11.2 (6.90 ± 2.00)	2 to 10.1 (6.80 ± 2.00)	91	124	80;80			(64, 66, 74)
IL-10	PBMCs	Post-stimulation (PGN, PHA, LPS, tetanus toxoid)	3	3.2 to 11.2 (6.90 ± 2.00)	2.7 to 10.1 (6.80 ± 2.00)	112	128	82;74			(66, 67, 74)
IL-5	PBMCs	Post-stimulation (PGN, PHA, LPS, tetanus toxoid)	3	3.2 to 11.2 (6.90 ± 2.00)	2.7 to 10.1 (6.80 ± 2.00)	112	128	82;74			(66, 67, 74)
IL-4	PBMCs	Post-stimulation (PGN, PHA, LPS, tetanus toxoid)	3	2.2 to 11.2 (6.90 ± 2.00)	2 to 10.1 (6.80 ± 2.00)	91	124	80;80			(64, 66, 74)

Symbols: ↑, increased; ↓, decreased; ≈, unchanged; in autism compared with controls.

ATP, adenosine triphosphate; BDE-47, 2,2’,4,4’-tetrabromodiphenyl ether; CD, cluster of differentiation; IFN-γ, interferon-γ; IL, interleukin; LPS, lipopolysaccharide; LTA, lipoteichoic acid; NK, natural killer; NR, not reported; PBMCs, peripheral blood mononuclear cells; PGN, polymeric peptidoglycan; PHA, phytohemagglutinin; PMA, phorbol myristate acetate; TNF-α, tumor necrosis factor-α.

**Table 3:** Percentages of blood immune cells in autism under baseline conditions compared with controls.

Cell type	Markers	No. of studies	Age range (in years) (mean ± SD)		Total sample size		% Males	Reported findings in autism compared with controls (in at least N = 3 references)		
			Autism	Controls	Autism	Controls	Autism; Controls	↑	↓	≈
T cells	CD3+	4	2.5 to 9 (4.25 ± 1.70)	2.2 to 9 (4.25 ± 2.20)	168	155	90;82			(65, 67, 75, 76)
T helper cells	CD4+	5	2.5 to 14.5 (5.21 ± 2.14)	2.2 to 11 (5.50 ± 2.06)	246	189	90;85			(22, 65, 67, 76, 77)
T suppressor cells	CD8+	5	2.5 to 14.5 (5.21 ± 2.14)	2.2 to 11 (5.50 ± 2.06)	210	180	91;84			(22, 65, 67, 76, 77)
Monocytes	CD14+, CD16+	5	2.5 to 14.5 (6.17 ± 2.58)	2.2 to 11 (6.75 ± 1.92)	213	138	87;82			(22, 45, 75, 77, 78)
B cells	CD19+	3	3 to 14.5 (6.17 ± 2.58)	3 to 11 (6.75 ± 1.92)	126	87	90;83			(22, 75, 78)
NK cells	CD3-CD56+, CD56+CD16+	3	3 to 56 (14.29 ± 3.09)	1 to 56 (13.9 ± 2.66)	189	96	83;76			(22, 72, 79)

Symbols: ↑, increased; ↓, decreased; ≈, unchanged; in autism compared with controls. CD, cluster of differentiation; NK, natural killer.

## Results

### Study characteristics

We identified a total of 3316 records in our search of six electronic databases: PubMed (N = 893), Scopus (N = 633), ProQuest Central (N = 1205), Ovid (N = 119), SAGE Journals (N = 52), and Wiley Online Library (N = 414), as illustrated in the PRISMA flow diagram in **Figure 1**. We screened the titles and abstracts of 2627 records after duplicate removal and identified 277 potentially relevant records. We retrieved the full-text articles of 277 records to assess eligibility according to our pre-defined inclusion and exclusion criteria. A total of 131 studies were excluded for the following reasons: non-original article (N = 11); non-autism subjects (N = 5); full-text articles not found (N = 2); non-English articles (N = 1); studies not pertaining to immune cells or cytokines or only on genetic DNA (N = 74); sample type not blood, plasma, or serum (N = 6); autism cases without controls (N = 16); studies on immune-related receptors, transcription factors, or activation markers (N = 5); and studies involving treatment or intervention (N = 11). The results of the quality assessments of the 146 eligible studies are listed in **Table S5**. Seventy-five studies had an overall rating of at least fair quality and were thus included in the qualitative data synthesis of this review.

### Data synthesis

We generated two comprehensive tables that summarize the reported findings from all 75 studies included in this review (**Table S3** and **S4**). Data related to immune cell percentages and cytokine production in immune cells are listed in **Table S3**, whereas data related to cytokine levels in the plasma, serum, or neonatal bloodspot are included in **Table S4**.

Of note, we did not include results from studies that were unrelated to cell quantification, such as activation markers, transcription factors, and immune cell receptors (e.g., IL-1Ra, CXCR3, CCR6, and IL-6sR). We also excluded results that were not related to cytokines, such as adhesion molecules (e.g., sPECAM-1, sVCAM-1, sP-selectin, and ICAM-1), and enzymes or enzyme inhibitors (e.g., thioredoxin, TIMP-1, and TIMP-2). Growth factors that are not cytokines, such as BDNF, GDNF, EGF, FGF2, NT-3, VEGF, PDGF-BB, and hepatocyte growth factor (HGH), were excluded from this review. Although we included “growth factor” in the search terms, this, in fact, refers to “transforming growth factor” (e.g., TGF-β) and “hematopoietic growth factors” (e.g., GM-CSF, G-CSF, IL-3), which are also cytokines. One study investigated immune responses in autism compared with controls and included other subgroups with and without gastrointestinal complications (66). As we were interested in results on autism as compared with controls in this review, we excluded results from the subgroups that included gastrointestinal complications (66).

Based on the results from **Tables S3** and **S4**, we identified findings that were consistently reported in at least three references. We generated three tables that focused on basal cytokine levels (**Table 1**), levels of cytokines produced by immune cells (**Table 2**), and the percentage of the immune cell population (**Table 3**) in autism relative to controls. A final list of the 62 studies, which formed the basis of this review, is included in **Tables 1**–**3** and described in the data summary section.

### Study population and sample analysis methods

We have included a detailed description of the study population and analysis methods in **Tables 1**–**3**, based on the data extracted from individual studies in **Supplementary Tables S3** and **S4**. Data related to the study population include age, total sample size, and sex, whereas data related to the analysis methods include sample type, *in vitro* stimulus, and analysis technique.

Overall, the mean age and age range of the study population in this review were comparable between the autism and control groups. The mean age ± standard deviation (SD) was 7.06 ± 0.70 and 7.17 ± 0.46 years for the autism and control groups, respectively. In **Table 1**, the age of subjects ranged from 2 to 12 years, apart from seven studies in which subjects reached 13, 14, 16, 21, and 28 years (22–24, 40, 46, 48, 49). Studies on MCP-1 and MIP-1α bloodspots were performed using neonatal samples (7, 8, 10, 11). In **Tables 2** and **3**, the age of subjects ranged from 2 to 12 years, except in a few studies in which the subjects’ ages reached 15, 17, and 56 years (**Table 2**) (55, 68, 72) and 14.5 and 56 years (**Table 3**) (22, 72). Regarding gender distribution, most of the studies in **Tables 1**–**3** involved the recruitment of male subjects (at least 70% of total subjects), which was similar between the autism and control groups. However, in a few studies, the overall percentage of male subjects only reached 67%, 64%, 59%, and 32% in the control group (**Tables 1** and **2**).

Meanwhile, the sample type was either plasma or serum for all cytokines included in **Table 1**, except the bloodspot samples used in the MCP-1 and MIP-1α analyses. The sample types were blood immune cells, including peripheral blood mononuclear cells (PBMCs), neutrophils, monocytes, T cells, and natural killer (NK) cells in **Table 2** and T cells, monocytes, B cells, and NK cells in **Table 3**. The techniques used in the analysis of the samples in **Table 1** were either ELISA or cytokine multiplex, as listed in **Supplementary Table S4**. The analysis techniques for samples in **Tables 2**–**3** include flow cytometry, ELISA, cytokine multiplex, qPCR, and western blot, as illustrated in **Supplementary Table S3**.

The stimuli used in the *in vitro* poststimulation studies are shown in **Table 2**. Different types of stimuli were used to assess cytokine production from various immune cells in autism, as compared with the control group. Studies on PBMCs and CD4+ T cells consistently showed differential responses from these cells in autism when stimulated with PMA/ionomycin and anti-CD3/CD28, respectively, as compared with the control group (61, 63, 69, 70). However, studies that used LPS, PHA, PGN or tetanus toxoid to stimulate PBMCs showed conflicting results, which might be due to differences in the type of cytokines analyzed in these studies (42, 64, 66, 67, 70, 71, 74). Therefore, the use of PMA/Ionomycin and anti-CD3/CD28 to stimulate PBMCs and CD4+ T cells, respectively, may represent a good model for studying cytokine production in autism. However, it should be noted that PMA/Ionomycin is a potent stimulant that bypasses receptor-mediated signal transduction mechanisms and directly activates intracellular signaling pathways that result in the production of various cytokines. Meanwhile, monocytes stimulated with LTA, neutrophils stimulated with IL-17A, and NK cells stimulated with IL-12/IL-18 or K562 cells showed differential responses in each of these cell types in autism, as compared with the controls (45, 56, 72, 73). However, the number of studies on these cell types is still limited, which makes it difficult to predict whether these stimuli can be effectively used for studying cytokine production in these cell types in autism.

### Data summary

In this review, we found that IL-6, IL-17, TNF-α, and IL-1β were increased in the in the serum but unchanged in the plasma in subjects with autism (**Table 1**). Interestingly, the increase in IL-6, IL-17, TNF-α, and IL-1β in the serum was in accordance with the elevated levels of these cytokines in *in vitro* stimulated neutrophils, monocytes, and CD4+ T cells in individuals with autism, as compared with controls (**Table 2**). Additionally, the levels of IL-6 and IL-17, but not TNF-α and IL-1β, were consistently higher in the unstimulated neutrophils, monocytes, and CD4+ T cells in autism subjects, as opposed to the controls (**Table 2**).

Meanwhile, studies have shown conflicting results for IFN-γ, as it is not only reported to be both increased and unchanged in plasma but also unchanged in the serum of subjects with autism (**Table 1**). This discrepancy in IFN-γ levels is also found in the response of blood immune cells after *in vitro* challenge, in which studies have delineated both increased and decreased IFN-γ levels in post-stimulated NK cells and CD4+ T cells, in addition to consistently increased levels in NK cells under baseline conditions (**Table 2**).

Likewise, reports on TGF-β are also conflicting, whereby it was found to be both increased and decreased in the plasma of autism subjects (**Table 1**), and little or no data pertaining to its cell of origin for autism are available. The two isoforms of TGF-β (β1 and β2) as well as the functional forms of TGF-β were treated equally during our data synthesis, which might have accounted for the variability observed in the TGF-β results (**Table 1**).

Studies have indicated an increase in the cytokine levels of RANTES and IL-8 relative to controls in subjects with autism (**Table 1**). In addition, reports consistently delineate unchanged IL-13, IL-10, IL-5, and IL-4 levels in the plasma/serum and post-stimulated blood immune cells in individuals with autism as compared with controls (**Tables 1** and **2**). Other cytokines, IL-12, IL-2, MCP-1, MIP-1α, GM-CSF, and eotaxin, were unchanged in the plasma of subjects with autism (**Table 1**).

**Figure 2:**
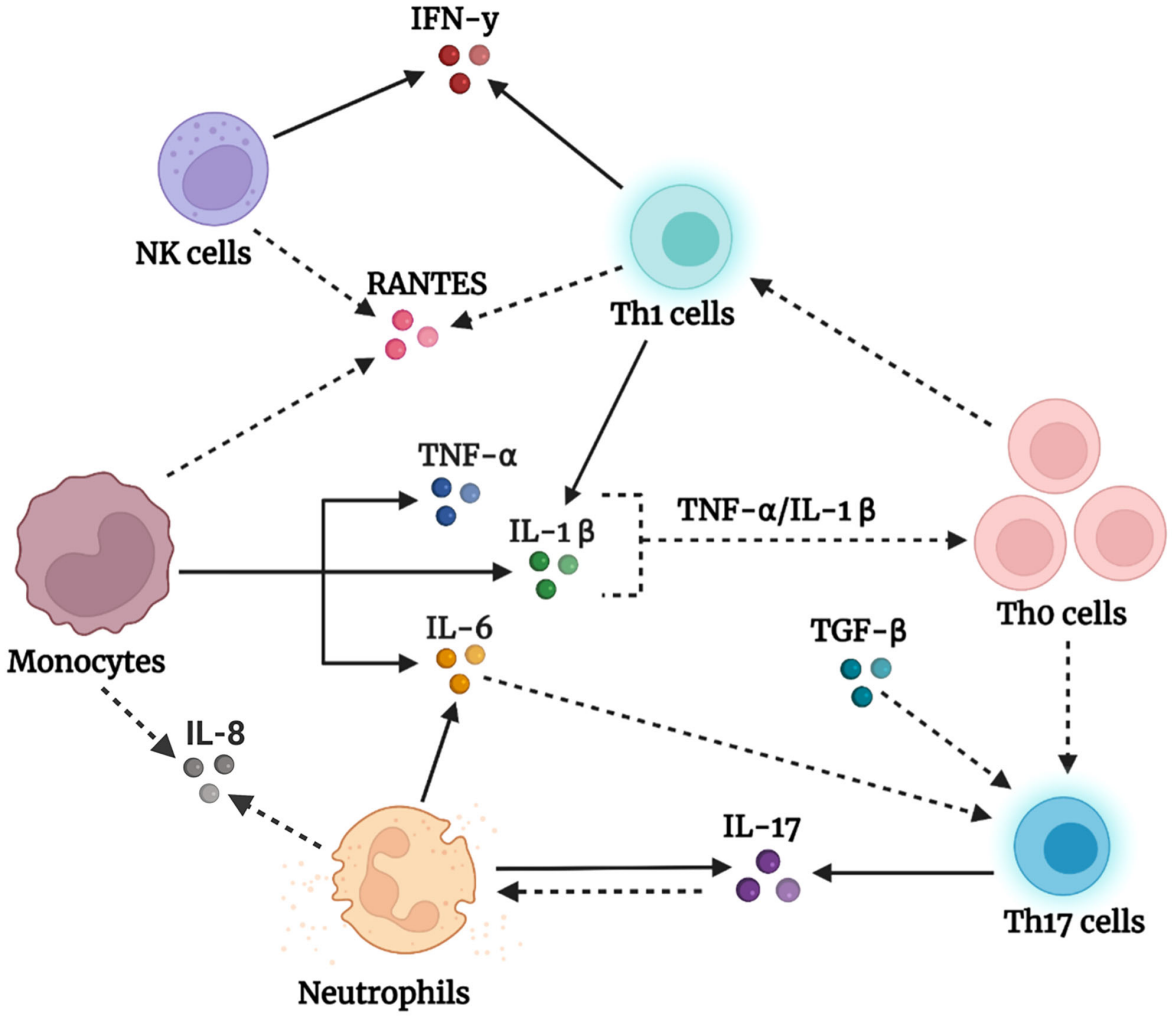
Schematic representation of our proposed model of peripheral immune dysregulation in autism.Activated monocytes produce high amounts of the triad of proinflammatory cytokines, TNF-α, IL-1β, and IL-6. Accordingly, TNF-α and IL-1β promote the activation, priming, and proliferation of naïve and effector T cells, whereas IL-6, in the presence of dysregulated levels of TGF-β, drives the polarization of naïve T helper (Th0) cells into effector Th17 subsets. The latter produces its signature cytokine IL-17, which initiates several events that result in neutrophil recruitment and activation (80). Neutrophils in individuals with autism produce IL-17 and IL-6, which add to the observed large pool of IL-17 and IL-6. In addition, naïve T helper (Th0) cells under the influence of cytokines in the milieu differentiate into Th1 cells, which impart high IFN-γ levels and enrich the IL-1β pool. Natural killer (NK) cells, on the other hand, tend to be dysfunctional in autism and chronically produce IFN-γ, which augments the IFN-γ pool. Furthermore, the high levels of the chemotactic factor RANTES might be derived from activated monocytes, T cells, or NK cells, whereas IL-8 might be produced by monocytes and neutrophils, and function to recruit cells to the scene. The solid arrows represent confirmed findings in the tables we formulated, whereas the dashed arrows are based on well-known facts in the literature that we suggest occur in the context of autism. Created using BioRender.com

Interestingly, the overall reported outcomes described above are unlikely to be caused by changes in the percentage of immune cells in subjects with autism compared with controls. As shown in **Table 3**, the frequency of CD3+ T cells and their subsets, CD4+ and CD8+ cells, as well as the frequency of monocytes, B cells, and NK cells were comparable between subjects with autism and controls. This might indicate that increased cytokine levels are not due to differences in immune cell numbers but rather due to the abnormal functional responses of these immune cells in individuals with autism as compared with controls. Based on the results in **Tables 1** and **2**, we present a schematic diagram illustrating our proposed model for the immune cells of origin of the altered cytokines in autism (**Figure 2**).

## Discussion

This systematic review was undertaken to investigate alterations in peripheral blood cytokines in autism and identify the immune cells contributing to abnormal cytokine profiles in individuals with this disorder. We focused on findings that showed a difference between autism and control subjects. Remarkably, we identified a list of specific cytokines that were altered in both plasma and serum, as well as in *in vitro* stimulated blood immune cells in autism. Collectively, we found increased levels of IL-6, IL-17, TNF-α, IL-1β, IFN-**γ**, RANTES, and IL-8 in the plasma/serum in autism, which is overall consistent with the findings from previous systematic reviews (18, 19). We reviewed autism studies that investigated cytokine production by *in vitro* stimulated blood immune cells and identified monocytes, neutrophils, CD4+ T cells, and NK cells as the major cellular sources of these altered cytokines. Conversely, we found TGF-β to be both increased and decreased in the plasma of patients with autism relative to controls, and there is as yet no conclusive evidence concerning its cellular sources. Interestingly, the immune cells identified in our review that produce certain cytokines in autism are also known to produce these cytokines. In this review, we also found that the percentage of the immune cell population is consistently unaltered in autism, suggesting that abnormal cytokine levels might be due to changes in the functionality or activation status of these cells rather than their numbers in autism.

In this review, we found elevated levels of IL-6, TNF-α, and IL-1β in the plasma/serum of individuals with autism. IL-6, TNF-α, and IL-1β are early proinflammatory cytokines released from immune cells in response to inflammatory stimuli. Importantly, these cytokines can cross from the peripheral blood into the brain, where they directly affect brain function and behavior (6, 16, 17). Previous studies have shown that high levels of IL-6, TNF-α, and IL-1β are correlated with the severity of autism symptoms, which include stereotypical behaviors and impaired communication skills (27, 33, 43, 45, 52). Interestingly, we found some evidence in our review for the potential cellular sources of these cytokines in autism. Elevated IL-6 levels have been described in monocytes and neutrophils at both baseline (56, 60) and after *in vitro* stimulation (45, 56) in autism, whereas augmented TNF-α levels have been described only in the post-stimulated monocytes of individuals with autism (45). In addition, IL-1β levels are markedly increased in post-stimulated monocytes and CD4+ T cells in autism (45, 70). Altogether, we suggest that monocytes are the predominant cells contributing to the elevated cytokine levels of IL-6, TNF-α, and IL-1β in autism, along with CD4+ T cells, which act as another source of IL-1β, whereas neutrophils are another source of IL-6. Previous studies have shown that TNF-α enhances T cell proliferation, augments IL-2R (interleukin-2 receptor) on T cells, and sustains T cell survival during the initiation of T cell responses (81). Similarly, IL-1β acts synergistically with IL-2 to promote the expansion of CD4 and CD8 T cells (82) and induces a robust and durable primary and secondary CD4 response (83). Therefore, we suggest that elevated TNF-α and IL-1β levels would promote naïve T helper (Th0) cell proliferation and expansion in the context of autism, as illustrated in our proposed model in **Figure 2**.

However, results for TGF-β are conflicting, whereby it is reported to be both increased (39, 46, 48) and decreased (35, 50, 51) in the plasma of individuals with autism, and there is scarce evidence concerning its cell of origin in autism. This discrepancy in TGF-β results might be due to differences between the studies in the isoforms or functional forms being assessed. TGF-β exists in three isoforms (β1, β2, and β3), which are structurally similar and share approximately 71%–79% sequence identity (84, 85). Although the three isoforms show similar functions *in vitro*, isoform-specific knockout mice show phenotypic differences, suggesting distinctive functions for these isoforms *in vivo* (84, 85). Unlike other cytokines that are produced in their active forms, TGF-β is secreted in an inactive form and stored in the extracellular matrix as a latent complex with its prodomain, which can then be converted into the active form through multiple mechanisms (86). In this review, studies showing decreased TGF-β levels specifically involved the TGF-β1 isoform (35, 50, 51), whereas increased levels of TGF-β were found for either the TGF-β2 or TGF-β1 isoform (39, 46, 48). Among these studies, only the functional form of TGF-β was reported in some studies, in which the activated form of TGF-β was specifically analyzed (35, 46, 50). Based on this evidence, we speculate that the activated TGF-β1 isoform is decreased, whereas the activated TGF-β2 isoform is increased in autism, which needs to be further investigated in future studies.

Given the dysregulated levels of TGF-β in autism, we speculate that this dysregulation may disrupt several immune processes in autism. TGF-β is an immunosuppressive cytokine produced by the immune system guardian-regulatory T (Treg) cells, which maintain immune homeostasis (49, 50) and are involved in diverse cellular functions, including the promotion of cell growth, proliferation, and differentiation (18). Alternatively, TGF-β can empower naïve T cell differentiation into effector T helper 17 (Th17) cells (87, 88), which are important for host defense and associated with inflammation and autoimmune conditions (89). This highly dynamic function of TGF-β is contextual; when it is produced in the presence of IL-6, TGF-β directs Th17 cell development and differentiation (90, 91) whereas when it is produced in the presence of IL-2, TGF-β promotes the development of induced regulatory T cells (92). Therefore, we suggest that dysregulated TGF-β levels, together with elevated IL-6 levels, may disrupt immune homeostasis in autism and affect Th17 cells, as shown in our proposed model in **Figure 2**.

In this review, we found evidence of increased IL-17 levels in the serum of subjects with autism (22, 33, 36). Interestingly, previous studies have shown that IL-17 signaling is responsible for autism-like symptoms in the offspring of maternal immune-activated mice (93, 94). From the studies we included, we identified neutrophils (56) and CD4+ T cells (62) as potential sources of IL-17 in the steady state of autism, in addition to IL-17 production by stimulated CD4+ T cells (62–64). IL-17 exerts important functions on T cells and neutrophils, including priming T cells and acting on neutrophils to promote the production of the proinflammatory cytokines IL-1, IL-6, and TNF-α (95). Although neutrophils are recruited to inflammatory sites under the influence of chemokines induced by IL-17 (80), they can become the main producers of IL-17 under certain circumstances, as in the case of kidney ischemia-reperfusion injury (96). The biological activity of IL-17 relies on the formation of multimeric receptors composed of IL-17RA and IL-17RC subunits (80). Interestingly, neutrophils of individuals with autism were found to express the IL-17RC subunit, which was absent in the neutrophils of control subjects. This enabled neutrophils to potently respond to IL-17A/IL-17R signaling and release IL-6 (56), leading to increased IL-17 production in autism. Taken together, these results suggest that Th17 cells and neutrophils are potential sources of upregulated IL-17 levels in autism (**Figure 2**).

Furthermore, we found increased levels of RANTES, IL-8, and IFN-**γ** in the plasma/serum of subjects with autism. However, there is still no clear evidence concerning the cellular sources of RANTES and IL-8 in our review. Given that RANTES is mainly produced by T cells, monocytes, and NK cells (97), we suggest that these cells also produce RANTES in autism (**Figure 2**), which does not exclude the possibility of the presence of other sources. Meanwhile, IL-8 is produced by monocytes and neutrophils, and we propose that these cell types in the circulating blood might also be potential sources of IL-8 in autism (**Figure 2**). In contrast, our review identified elevated production of IFN-**γ** in *in vitro* stimulated CD4+ T cells (70) and NK cells in the basal state in autism (72, 73). However, reduced levels of IFN-γ were observed in NK cells in autism after introducing an external stimulus, such as IL-12/IL-18 or K562 target cells (72, 73). These observations suggest that NK cells in autism are persistently activated and produce abnormal amounts of IFN-γ under baseline conditions; however, they tend to be hypofunctional (exhausted) after an *in vitro* immune challenge. Interestingly, the same NK cell phenotype has also been reported in other psychiatric disorders, including obsessive-compulsive disorder, chronic stress, and depression (98, 99). Future studies are needed to further investigate the exhausted NK cell phenotype in autism, where this phenotype might be representative of a subpopulation of subjects with autism.

It is worth mentioning that the studies presented in this review suggest the prevalence of T helper 1 (Th1) and Th17 phenotypes rather than the Th2 phenotype in autism. CD4+ T helper (Th) cells are subdivided into different subsets distinguished by their unique transcription factors and cytokine production. These subsets include IFN-γ-producing Th1 cells, IL-4-producing Th2 cells, IL-17-producing Th17 cells, and Foxp3-expressing Treg cells. Importantly, T helper cell differentiation toward a specific subset actively inhibits the transcriptional program of the other subset and its ability to produce characteristic cytokines (100). Therefore, our findings indicate that increased production of Th1 cytokines (IFN-γ) and Th17 cytokines (IL-17), along with no change in Th2 cytokines (IL-4, IL-5, and IL-13), might suggest a skewing toward the Th1/Th17 phenotype and dampening of the Th2 phenotype in the context of autism (**Figure 2**). Interestingly, Th1 cytokines were shown to be associated with autism severity and impaired behavior, whereas Th2 cytokines were correlated with better behavioral and developmental scores (67).

In addition, there are some limitations that should be considered in our systematic review. We restricted our search to articles written in English only and, thus, may have neglected results from articles in other languages. Other limitations include differences in the age range of subjects among the different studies, in addition to technical variations, such as the use of different analysis methods, sample types, and *in vitro* stimuli, which together might have contributed to the variability in the observed results across studies. The overall age range of subjects from the studies included in this review was between 2 and 12 years; however, it is important to emphasize that the immune system undergoes dynamic changes during this developmental period from early childhood to adolescence (101), which might introduce some variability when combining results from different studies. Therefore, future studies should consider restricting the age range of subjects for a more comprehensive understanding of immune changes in autism.

In our review, we also observed differences in some cytokines when measured in serum versus plasma samples, which is also supported by previous studies (102–104). This inconsistency in the results between plasma and serum might be attributed to differences in the preparation methods for these sample types. Serum samples are obtained after blood coagulation, whereas the preparation of plasma samples involves the use of various anticoagulants that affect cytokine measurement (102–104). Although the studies included in this review have used standard methods for cytokine detection, such as ELISA, cytokine multiplex, or flow cytometry, significant differences might still exist regarding sample handling and processing methods, the analysis platforms used, and the sensitivity and specificity of the antibodies used across different studies. In addition, there are variations in the type of *in vitro* stimuli, such as LPS, PHA, or PGN, used in PBMCs culture models, which could lead to variations in cell responses.

Currently, there are a limited number of studies investigating cytokine production at the level of a specific immune cell type in autism, and much of the present evidence pertains to total PBMCs. Although we found consistent results regarding the type of altered cytokines produced by blood immune cells in autism, these results were not representative of a specific immune cell type and may include results from PBMCs. In addition, the results presented in this review and the proposed model are based on currently available evidence from the autism literature; therefore, it does not exclude the fact that other cellular sources, such as innate lymphoid cells and NK T cells, although present in very small numbers in the circulating blood, might also contribute to the production of IL-17, IFN-**γ**, and other T cell cytokines, which have yet to be investigated. Furthermore, endothelial cells and tissue-resident cells are other potential sources for cytokine production, however we focused in our review on peripheral immune cells in the circulating blood in autism, which are less invasive and have current evidence in the literature. Finally, most of the studies included in this review were cross-sectional in nature and lacked details of the clinical features and comorbid conditions of autism subjects, which can be considered other limitations of the current studies in the field. These highlight the need for more longitudinal analyses in future studies in a well-characterized study population for more accurate detection of time-dependent changes in immune phenotypes in autism and their correlation with clinical and behavioral changes in autism.

## Conclusions

This systematic review supports the existing evidence of abnormal cytokine levels in the peripheral blood of individuals with autism. Given that cytokines are ubiquitous molecules circulating in the bloodstream, determining their cellular sources is crucial for targeted therapeutics. To our knowledge, this is the first review to identify the potential immune cells of origin of the altered cytokines in autism, and we propose a network model that integrates these altered cytokines and their specific cellular sources in autism. Although certain areas presented in this review still lack solid evidence, this review summarizes the current knowledge in the field and highlights the gaps that need to be considered in future studies. Most current studies on autism have focused on cytokine levels in the blood or analyzed cytokine production from a population of PBMCs. We emphasize the importance of future studies on autism to further elucidate immune sources of altered cytokines at the single-cell level and combine multi-omics approaches, such as proteomics, cell phenotyping, and single-cell transcriptomic analyses, which may reveal cell-specific immune pathways underlying autism. Since autism is a heterogeneous condition, immune phenotypes may vary widely within a group of subjects with autism. Future studies should consider the utilization of advanced bioinformatics and machine learning techniques to segregate autism groups into different subgroups based on their exhibited immune and clinical phenotypes, which might also lead to the identification of novel characteristics of subclusters of subjects with autism for a better understanding of the pathophysiology of autism.

## Data availability statement

The original contributions presented in the study are included in the article/**Supplementary Material**. Further inquiries can be directed to the corresponding author.

## Author contributions 

AA-S conceived the review. WN-E, SL, NM, and AA-S collected and summarized the review data. WN-E and AA-S wrote the manuscript. All authors contributed to the article and approved the submitted version.

## Funding

This publication was made possible by ECRA Award number ECRA01-001-3-001 from the Qatar National Research Fund (a member of Qatar Foundation), and start-up fund from Qatar Biomedical Research Institute (Grant code VR03).

## Acknowledgments

We would like to thank Editage (www.editage.com) for English language editing.

## Conflict of interest

The authors declare that the research was conducted in the absence of any commercial or financial relationships that could be construed as a potential conflict of interest.

## Publisher’s note

All claims expressed in this article are solely those of the authors and do not necessarily represent those of their affiliated organizations, or those of the publisher, the editors and the reviewers. Any product that may be evaluated in this article, or claim that may be made by its manufacturer, is not guaranteed or endorsed by the publisher.

## Author disclaimer

The findings achieved herein are solely the responsibility of the authors.
