# Supplementary material for: In search of immune cellular sources of abnormal cytokines in the blood in autism spectrum disorder: A systematic review of case-control studies
Source: Front Immunol. 2022 Oct 4;13:950275. doi: 10.3389/fimmu.2022.950275 (PMC9578337; doi:10.3389/fimmu.2022.950275)
Supplement: Supplementary file 1 [file DataSheet_1.zip › Supplementary material/Table S2.pdf]

Table S2. List of search terms and applied filters in each of the electronic databases used in this systematic review.

Database	Search terms	Applied filters
**PubMed**	**(under Advanced Query box; All Fields):**<br>(autism OR autistic OR ASD) AND (human OR subject OR child OR participant OR patient OR volunteer) AND (blood OR plasma OR serum OR immune OR peripheral OR circulating) AND ((cytokine OR chemokine OR "growth factor" OR interferon OR "tumor necrosis factor" OR "colony stimulating factor" OR interleukin) OR (lymphocyte OR monocyte OR "B cell" OR "T cell" OR "natural killer" OR "dendritic cell" OR neutrophil OR basophil OR eosinophil OR myeloid))	**Article Type:** Journal Article<br>**Language:** English
**Scopus**	**(under Documents):**<br>• **Keywords:** (Autism OR autistic OR ASD)<br>• **AND Title-Abstract-Keyword:** (human OR subject OR child OR participant OR patient OR volunteer)<br>• **AND Title-Abstract-Keyword:** (blood OR plasma OR serum OR immune OR peripheral OR circulating)<br>• **AND Title-Abstract-Keyword:** (cytokine OR chemokine OR "growth factor" OR interferon OR "tumor necrosis factor" OR "colony stimulating factor" OR interleukin)<br>• **OR Title-Abstract-Keyword:** (lymphocyte OR monocyte OR "B cell" OR "T cell" OR "natural killer" OR "dendritic cell" OR macrophage OR neutrophil OR basophil OR eosinophil OR myeloid)	**Limit to Document Type:** Article<br>**Source Type:** Journal<br>**Language:** English
**ProQuest Central**	**(under Advanced in Anywhere):**<br>(autism OR autistic OR ASD) AND (human OR subject OR child OR participant OR patient OR volunteer) AND (blood OR plasma OR serum OR immune OR peripheral OR circulating) AND ((cytokine OR chemokine OR "growth factor" OR interferon OR "tumor necrosis factor" OR "colony stimulating factor" OR interleukin) OR (lymphocyte OR monocyte OR "B cell" OR "T cell" OR "natural killer" OR "dendritic cell" OR neutrophil OR basophil OR eosinophil OR myeloid))	**Database:** Include (Health & Medical Collection)<br>**Subject:** Include (autism); Exclude NOT (animals AND rodents AND animal models)<br>**Source type:** Scholarly Journals<br>**Document type:** Article<br>**Language:** English
**Ovid**	**(under Multi-Field Search):**<br>• **Author Keywords:** (Autism OR autistic OR ASD)<br>• **AND All Fields:** (human OR subject OR child OR participant OR patient OR volunteer) AND (blood OR plasma OR serum OR immune OR peripheral OR circulating) AND ((cytokine OR chemokine OR "growth factor" OR interferon OR "tumor necrosis	**Limits selected:** Original Articles; English Language<br>**Resource selected:** Your Journals@Ovid; and Ovid MEDLINE(R) ALL 1946 to July 07, 2020

	factor" OR "colony stimulating factor" OR interleukin) OR (lymphocyte OR monocyte OR "B cell" OR "T cell" OR "natural killer" OR "dendritic cell" OR macrophage OR neutrophil OR basophil OR eosinophil OR myeloid))	
**SAGE Journals**	**(under Refine Search):**<br>• **Keywords:** (Autism OR autistic OR ASD)<br>• **AND Anywhere:** (human OR subject OR child OR participant OR patient OR volunteer) AND (blood OR plasma OR serum OR immune OR peripheral OR circulating) AND ((cytokine OR chemokine OR "growth factor" OR interferon OR "tumor necrosis factor" OR "colony stimulating factor" OR interleukin) OR (lymphocyte OR monocyte OR "B cell" OR "T cell" OR "natural killer" OR "dendritic cell" OR macrophage OR neutrophil OR basophil OR eosinophil OR myeloid))	**Selected:** Research Articles
**Wiley Online Library**	**(under Advanced):**<br>• **Keywords:** (Autism OR autistic OR ASD)<br>• **AND Anywhere:** (human OR subject OR child OR participant OR patient OR volunteer) AND (blood OR plasma OR serum OR immune OR peripheral OR circulating) AND ((cytokine OR chemokine OR "growth factor" OR interferon OR "tumor necrosis factor" OR "colony stimulating factor" OR interleukin) OR (lymphocyte OR monocyte OR "B cell" OR "T cell" OR "natural killer" OR "dendritic cell" OR macrophage OR neutrophil OR basophil OR eosinophil OR myeloid))	**Publication Type:** Journals
