# Supplementary material for: In search of immune cellular sources of abnormal cytokines in the blood in autism spectrum disorder: A systematic review of case-control studies
Source: Front Immunol. 2022 Oct 4;13:950275. doi: 10.3389/fimmu.2022.950275 (PMC9578337; doi:10.3389/fimmu.2022.950275)
Supplement: Supplementary file 1 [file DataSheet_1.zip › Supplementary material/Table S3 and S4.pdf]

Table S3. Summary of the characteristics and findings of studies investigating the percentages of immune cells as well as cytokine levels produced or expressed by immune cells of individuals with autism compared to typically developing individuals.

Age range (in years) (mean ± SD; median)		Samples size (N)		Sample type	Analysis techniques	Reported findings in autism compared to controls Results in baseline, stimuli between brackets [ ] if used			Ref.
Autism	Controls	Autism	Controls			↑	↓	≈	
3 to 15	4 to 12	37	40	PBMCs	qPCR	IL-6			(1)
3.17 to 4.25	3 to 4.50	34	26	PBMCs	ELISA, flow cytometry		IL-23 [PHA]	IL-17 [PHA]<br><br>T cells, B cells, monocytes (data and markers not shown), Th17 cells [PMA]	(2)
2 to 6	2 to 6	45	69	PBMCs	Cytokines multiplex	IL-17 [PHA]		IL-17, IL-13, IL-4<br><br>IL-13, IL-4 [PHA]	(3)
3.5 to 10.7	3.4 to 10.1	20	20	PBMCs	ELISA, complete blood count	IL-4, IL-5, IL-13<br><br>Eosinophil count		IL-2, IL-4, IL-5, IL-10, IL-13, IFN-γ [PHA, tetanus toxoid, house dust mite antigen]	(4)
3.1 to 4.5 (3.6)	2.5 to 4.0 (3.3)	37	35	PBMCs	Cytokines multiplex, flow cytometry	GM-CSF, TNF-α, IL-13 [PHA]	IL-12p40 [PHA]<br><br>IFN-γ [tetanus toxoid]	IL-5, IL-6, IL-8, IL-10 [PHA, tetanus toxoid]<br><br>CD3+, CD4+, CD8+ cells	(5)
2.2 to 5	2.3 to 4.8	17	16	CD3-CD14+ monocytes	Cytokines multiplex, flow cytometry	IL-1β [LTA, LPS]<br><br>IL-6, TNF-α [LTA]	MCP-1 [polyI:C, LPS]<br><br>IL-1β, IL-6, TNF-α, GM-	IL-8 [LTA, polyI:C, LPS, flagellin, CpG-B]<br><br>CD14+ monocytes	(6)

Age range (in years) (mean ± SD; median)		Samples size (N)		Sample type	Analysis techniques	Reported findings in autism compared to controls Results in baseline, stimuli between brackets [ ] if used			Ref.
**Autism**	**Controls**	**Autism**	**Controls**			↑	↓	≈	
							CSF, MCP-1 [CpG-B]		
3 to 12 (4.99 ± 1.34)	3 to 12 (5.05 ± 1.53)	35	25	CD45+ PBMCs	Flow cytometry, qPCR, western blot	GM-CSF, IFN-γ, IL-6, IL-9, IL-22 [PMA/ionomycin]			(7)
(11.6 ± 2.7)	(11.1 ± 2.3)	30	30	PBMCs	qPCR		TNF-α		(8)
3 to 12 (7.83 ± 2.73)	3 to 12 (7.55 ± 2.44)	53	28	PBMCs, CD4+, CD8+ T cells, CD14+ monocytes	Flow cytometry, qPCR, western blot	IL-16 in PBMCs, CD4+, CD8+, CD14+ cells [PMA/ionomycin]			(9)
3 to 11 (7.69 ± 2.26)	3 to 11 (7.76 ± 2.45)	50	45	PBMCs, CD4+, CD14+ cells	Flow cytometry, qPCR, western blot	IL-21, IL-22 in PBMCs and CD4+ T cells [PMA/ionomycin]	IL-27 in PBMCs and CD14+ cells [PMA/ionomycin]		(10)
2.42 to 5 (3.42)	2.5 to 4.75 (3.5)	19	18	PBMCs	Cytokines multiplex	IL-1β, IL-8 [LPS+BDE-47]			(11)
2 to 10 (4.33)	2 to 17 (5)	31	21	PBMCs	qPCR	TNF-α, IFN-γ, IL-8, IL-10		IL-1β, IL-6	(12)
2 to 5 (3.43 ± 1.03)	2 to 5	64	64	Whole blood	Complete blood count	Neutrophil counts	Platelet and lymphocyte counts		(13)

Age range (in years) (mean ± SD; median)		Samples size (N)		Sample type	Analysis techniques	Reported findings in autism compared to controls Results in baseline, stimuli between brackets [ ] if used			Ref.
**Autism**	**Controls**	**Autism**	**Controls**			↑	↓	≈	
(7.6 ± 1.8)	(7.2 ± 1.5)	38	32	CD14+ monocytes	Flow cytometry, qPCR	IL-6, IL-1β			(14)
(6.6 ± 1.4)	(6.9 ± 1.6)	30	25	CD4+ T cells	Flow cytometry, ELISA	IL-17A [anti-CD3/CD28]			(15)
(6.7 ± 1)	(6.5 ± 1)	45	35	CD4+ T cells	Flow cytometry, ELISA	IL-17A<br><br>IL-17A [PMA/Ionomycin]<br><br>IL-17A [IL-6]			(16)
5.46 to 11.23 (7.83)	5.50 to 8.33 (6.75)	26	35	PBMCs	Cytokines multiplex	IL-1α, IL-1β, TNF-α [LPS]<br><br>IL-15 [PHA]	IL-15, IL-17, IL-12p70 [LPS]<br><br>IL-1α, IL-1β, IL-6, IL-12p40, TNF-α, IFN-γ [PGN]	IL-4, IL-13, IL-10, TGF-β1, IL-5 [LPS, PGN, PHA]	(17)
3 to 11 (7)	3 to 11 (7)	25	30	PBMCs	qPCR, ELISA	IL-1β, IL-18, IL-37, IL-6, GRO-A, GRO-B [LPS+ATP]	IL-33 [LPS+ATP]		(18)
(13.51 ± 4.21)	(11.90 ± 3.73)	45	43	Monocytes	Complete blood count	Monocytes count			(19)

Age range (in years) (mean ± SD; median)		Samples size (N)		Sample type	Analysis techniques	Reported findings in autism compared to controls Results in baseline, stimuli between brackets [ ] if used			Ref.
Autism	Controls	Autism	Controls			↑	↓	≈	
4 to 6 (5.35)	4 to 6 (5.7)	70	35	Whole blood	Micro-volume laser scanning cytometry	CD20+ B cells, CD5- mature B cells, CD56+CD3- NK cells		CD4+ T cells, CD8+ T cells, monocytes, neutrophils, eosinophils, platelets (Markers not clearly mentioned)	(20)
4 to 12 (8.27 ± 2.66)	4 to 12 (8.03 ± 2.5)	30	30	Regulatory T (Treg) cells	Flow cytometry		CD4+ $CD25^{high}$ Treg cells		(21)
3 to 22 (6.7 ± 3.6)	1 to 12 (5.2 ± 3.4)	104	31	Natural killer (NK) cells	Flow cytometry		CD57+CD-NK cells	CD56+CD3- NK cells	(22)
3.5 to 4.9 (4.25 ± 1.70)	3.3 to 5.1 (4.25 ± 2.20)	24	24	B cells, T cells, NK cells	Flow cytometry	CD19+CD23+ B cells		CD3+, CD4+, CD8+, NK (No marker), CD19+, CD4+CD25+ Treg cells	(23)
2.8 to 3.75 (2.92 ± 0.05)	2.7 to 3.58 (2.96 ± 0.06)	57	29	Myeloid dendritic cells (mDCs), plasmacytoid DCs (pDCs)	Flow cytometry	Lin1−BDCA1+CD11c+ (mDCs), Lin1−BDCA3+CD123− (mDCs)		Lin1−BDCA2+CD123+ (pDCs), Lin-1−BDCA4+ CD11c− (pDCs)	(24)
2.2 to 5.6 (3.7)	2.3 to 4.8 (3.4)	52	27	CD56+ CD3- CD8- NK cells	Flow cytometry, qPCR	CD56+ NK cells<br><br>IFN-γ	IFN-γ [K562 cells]		(25)

Age range (in years) (mean ± SD; median)		Samples size (N)		Sample type	Analysis techniques	Reported findings in autism compared to controls Results in baseline, stimuli between brackets [ ] if used			Ref.
Autism	Controls	Autism	Controls			↑	↓	≈	
2.5 to 4.8 (3.6)	2.2 to 6.1 (3.9)	36	27	CD3+, CD3+CD8+, CD3+CD4+ T cells	Flow cytometry, ELISA			TNF-α<br>CD3+, CD4+, CD8+ T cells<br><br>TNF-α [PHA]<br>CD3+, CD4+, CD8+ T cells [PHA]	(26)
3 to 14.5 (6.17 ± 2.58)	3 to 11 (6.75 ± 1.92)	50	30	T cells, monocytes, NK cells, DCs, B cells	Flow cytometry	HLADR+CD11c+ myeloid DCs		CD8+ T cells, CD4+ T helper (Th) cells, and subsets: CXCR3+CCR6- Th1, CXCR3-CCR6- Th2, CXCR3-CCR6+ Th17, CD25+CD127^{dim} Tregs, and further subsets: CCR4+CD45RO^{neg} naïve Tregs, CCR4+CD45RO+ memory Tregs<br><br>CD14+ monocytes, and subset CD16+ non-classical monocytes<br><br>CD16+CD56+ NK cells<br><br>CD16-HLADR+ DCs and subsets CD123+ plasmacytoid DCs,	(27)

Age range (in years) (mean ± SD; median)		Samples size (N)		Sample type	Analysis techniques	Reported findings in autism compared to controls Results in baseline, stimuli between brackets [ ] if used			Ref.
Autism	Controls	Autism	Controls			↑	↓	≈	
								CD11c+ myeloid DCs<br><br>CD19+ B cells and subsets: CD20-CD27+CD38+ plasmablasts, CD20+CD24+CD38+ transitional cells, CD20+CD27+ memory B cells, CD20+CD27-IgD- naïve B cells	
18 to 56 (30)	Age matched to autism (but no range mentioned)	35	35	CD3-CD56+ NK cells	Flow cytometry	IFN-γ	IFN-γ [IL-12+IL-18]	CD4-CD56+ NK cells	(28)
5.08 to 9 (6.83)	4 to 9 (6.08)	42	31	PBMCs, B cells, T cells, monocytes	Flow cytometry, ELISA			CD19+ B cells, CD3+ T cells, CD14+ monocytes, CD19+CD27−IgD+ naïve B cells, CD19+CD27+IgM+ memory B cells, or CD19+CD27+IgG+ memory B cells	(29)
(6.8)	(7.6)	21	20	PBMCs	qPCR	IL-6		IL-10, IL-12A, IL-1β, TNF-α	(30)

Age range (in years) (mean ± SD; median)		Samples size (N)		Sample type	Analysis techniques	Reported findings in autism compared to controls Results in baseline, stimuli between brackets [ ] if used			Ref.
**Autism**	**Controls**	**Autism**	**Controls**			↑	↓	≈	
3 to 11 (6.92 ± 2.7)	3 to 11 (7.22 ± 2.72)	40	40	PBMCs, CD4+ T cells	Flow cytometry, qPCR, western blot	IL-1β, IL-4, IFN-γ, IL-9 in PBMCs and CD4+ cells [anti-CD3/CD28]			(31)
(7.4 ± 3.25)	(7.6 ± 2.90)	45	40	CD16+ Neutrophils	Flow cytometry, ELISA, qPCR	IL-17A, IL-6<br><br>IL-6 [IL-17A]			(32)
(7.0 ± 3.8)	(9.2 ± 4.0)	195	162	PBMCs	qPCR	IL-6	IL-17	IL-1β, IL-1α, IL-4, TNF-α, TGF-β	(33)
3 to 11 (7.65 ± 2.1)	3 to 11 (7.91 ± 2.22)	40	40	PBMCs	Flow cytometry, qPCR, western blot	IL-1β, IFN-γ [PMA/ionomycin]<br><br>CD11a, CD11b, CD14 [PMA/ionomycin]			(34)
(6 ± 1.4)	(6 ± 1.4)	30	41	Whole blood	qPCR	TNF-α, IL-6, IL-17	IL-2	TGF-β, IFN-γ, IL-8, IL-1β, IL-4	(35)

Table S4. Summary of the characteristics and findings of studies investigating cytokine levels in the serum, plasma or bloodspots of individuals with autism in comparison to typically developing individuals.

Age range (in years) (mean ± SD; median)		Sample size (N)		Sample type	Analysis techniques	Reported findings in autism compared to controls			Ref.
Autism	Controls	Autism	Controls			↑	↓	≈	
18 to 28 (22.7±2.26)	18 to 26 (23.4 ± 2.61)	19	21	Serum	ELISA		TGF-β1		(36)
3 to 4.2 (3.4)	2.8 to 4.5 (3.6)	75	36	Plasma	ELISA		TGF-β1		(37)
3 to 11 (7.69 ± 2.26)	2 to 11 (7.76 ± 2.45)	42	42	Serum	ELISA	Osteopontin			(38)
3 to 4.5 (3.6)	2.8 to 4.3 (3.6)	80	58	Plasma	Human bead immunoassays	MCP-1, RANTES, Eotaxin		MIP-1α, MIP-1β, IP-10	(39)
5 to 10 (7.77 ± 1.66)	5 to 10 (7.60 ± 1.77)	99	40	Plasma	Cytokines multiplex		IL-1α, IL-6, G-CSF, Fractalkine, MCP3, MIP1α, MIP1β	IL-1β, IL-1Rα, IL-2, IL-2Rα, IL-9, IL-12p40, IL-12p70, IL-17, Eotaxin, GM-CSF, GRO, IL-8, IP-10, MCP1, MDC, TGF-α, TNF-α, IFN-α2, IFN-γ	(40)
4 to 11 (6.9 ± 1.9)	5 to 12 (7.08 ± 1.78)	62	62	Serum	ELISA	ENA-78			(41)
4 to 12 (7.54 ± 1.96)	5 to 11 (7.19 ± 1.69)	56	32	Serum	ELISA	MDC, TARC			(42)
2.9 to 4.3 (3.4)	2.8 to 4.1 (3.4)	97	87	Plasma	Cytokines multiplex	IL-1β, IL-6, IL-8, IL-12p40		IL-2, IL-4, IL-5, IL-10, IL-13, GM-CSF, IFN-γ, TNF-α	(43)

Age range (in years) (mean ± SD; median)		Sample size (N)		Sample type	Analysis techniques	Reported findings in autism compared to controls			Ref.
Autism	Controls	Autism	Controls			↑	↓	≈	
Neonates	Neonates	214	62	Bloodspot	Cytokines multiplex	IL-1β, IL-4		IL-2, IL-5, IL-6, IL-10, IL-12, IL-13, IFN-γ, TNF-α, IL-8, MCP-1, MIP-1α, MIP-1β, IP-10, RANTES	(44)
Neonates	Neonates	84	159	Bloodspot	Cytokines multiplex	MCP-1	RANTES	MIP-1α, Eotaxin	(45)
Neonates	Neonates	414	820	Bloodspot	Cytokines multiplex		TGF-β		(46)
Neonates	Neonates	359	741	Bloodspot	Cytokines multiplex		RANTES	MIP-1α, MCP-1	(47)
Neonates	Neonates	359	741	Bloodspot	Cytokines multiplex	IL-8	IFN-γ, IL-2, IL-12, IL-4, IL-5, IL-6, IL-10, GM-CSF, IL-1β	IL-17, IL-18, TNF-α, TNF-β,	(48)
(5.51±1.28)	(6.11±1.04)	32	28	Serum	Cytokines multiplex	TNF-α, IL-1β, IL-17A		IL-17F, IFN-γ, IL-10, IL-22, IL-2, IL-21, IL-4, IL-6	(49)
4 to 12 (7.72 ± 2.65)	4 to 12 (8.4 ± 2.66)	30	30	Serum	ELISA	TNF-α			(50)

Age range (in years) (mean ± SD; median)		Sample size (N)		Sample type	Analysis techniques	Reported findings in autism compared to controls			Ref.
Autism	Controls	Autism	Controls			↑	↓	≈	
Neonates	Neonates	370	378	Bloodspot	Cytokines multiplex with 2 single-plex	IL-6, IL-8		6CKINE, BCA-1, CTACK, ENA-78, Eotaxin-1, Eotaxin-2, Eotaxin-3, Fractalkine, GCP-2, GM-CSF, GRO-A, GRO-B, I-309, IFN-γ, IL-1B, IL-2, IL-4, IL-10, IL-16, IP-10, I-TAC, MCP-1, MCP-2, MCP-3, MCP-4, MDC, MIF, MIG, MIP-1A, MIP-1D, MIP-3A, MIP-3B, MPIF-1, SCYB16, SDF-1A-B, TARC, TECK, TNF-α, IL-12p70, IL-13	(51)
2 to 10 (4.7 ± 2.4)	2 to 10 (5.4 ± 2.4)	35	35	Serum	ELISA	IL-1β, IL-8, TNF-α			(52)
6 to 11 (8.44 ± 1.73)	6 to 11 (8.33 ± 1.72)	45	40	Serum	ELISA	IL-17A			(53)
(9.71 ± 4.99)	(8.36 ± 2.85)	30	18	Plasma	ELISA			IL-33, IL-1β	(54)
1.58 to 6.17 (3.33)	3.33 to 6.58 (4.58)	87	41	Plasma	Cytokines multiplex	Eotaxin, TGF-β1, TNF-α		Fractalkine, IFN-γ, IL-8, MCP-1, MIP-1α, MIP-1β	(55)
3 to 13	3 to 13	30	30	Plasma	ELISA		TGF-β1		(56)

Age range (in years) (mean ± SD; median)		Sample size (N)		Sample type	Analysis techniques	Reported findings in autism compared to controls			Ref.
Autism	Controls	Autism	Controls			↑	↓	≈	
4 to 12 (6.97 ± 1.67)	4 to 12 (7.81 ± 2.73)	21	21	Serum	ELISA	IL-1β, IL-6			(57)
3 to 16	3 to 16	20	19	Plasma	ELISA	TGF-β2, IFN-γ			(58)
4 to 12	4 to 11	20	19	Plasma	ELISA	IFN-γ	TNF-α, IL-6		(59)
2.08 to 4.83	2.58 to 5.17	40	20	Plasma	ELISA		IL-23	IL-17	(60)
(7.13 ± 3.89)	(6.75 ± 3.96)	40	35	Plasma	ELISA, cytokines multiplex	TNF-α		IL-1β, IL-4, IL-6, IL-10, IL-17A, IFN-γ	(61)
2 to 12	2 to 14	38	32	Plasma	ELISA	TGF-β2			(62)
6.1 to 11.8 (8.7 ± 1.2)	6.3 to 12 (9.1 ± 1.7)	50	50	Plasma	ELISA		TGF-β1, IL-23	IL-17	(63)
3 to 10 (6 ± 1.75)	5 to 12 (7.35 ± 1.93)	42	20	Serum	ELISA	IL-1β, IL-4, IL-6, IL-13		IL-8, IL-10, TNF-α, IFN-γ	(64)
< 15 (8.11 ± 3.65)	< 15 (7.44 ± 3.12)	25	25	Plasma	Multiplex sandwich ELISA			BCA-1, Eotaxin, Eotaxin-2, G-CSF, GM-CSF, I-309, IFN-γ, IL-1α, IL-1β, IL-2, IL-4, IL-5, IL-6, IL-7, IL-8, IL-10, IL-11, IL-12p40, IL-12p70, IL-13, IL-15, IL-16, IL-17, MCP-1, M-CSF, MIG, MIP-1α, MIP-1β, MIP-1δ,	(65)

Age range (in years) (mean ± SD; median)		Sample size (N)		Sample type	Analysis techniques	Reported findings in autism compared to controls			Ref.
Autism	Controls	Autism	Controls			↑	↓	≈	
								RANTES, TNF-α, TNF-β	
(4.5 ± 1.3)	(4.5 ± 1.3)	102	102	Serum	ELISA	MIF, IL-6			(66)
2 to 21	Age matched to autism (but no range mentioned)	29	29	Serum	ELISA	IL-1β, IL-6, IL-12, IL-23, TNF-α		IL-13	(67)
5.3 to 10	5.6 to 8.4 (7)	30	39	Plasma	Multiplex		IL-35		(68)
3.5 to 10.5 (4.8 ± 2.4)	3 to 9.5 (4.2 ± 1.9)	80	60	Serum	EASIA	IL-1β			(69)
3 to 8 (4.51 ± 0.69)	Age matched to autism (4.06 ± 0.86)	45	38	Plasma	Cytokines multiplex	IFN-α, IL-7, IL-8, MIP-1α, MIP-1β, RANTES	IL-1β, IL-2, IP-10	Eotaxin, GM-CSF, IFN-γ, IL-10, IL-12p40/p70, IL-13, IL-15, IL-17, IL-1RA, IL-2R, IL-4, IL-5, IL-6, MCP-1, MIG, TNF-α	(70)
3.8 to 6.0 (4.29 ± 0.97)	3.7 to 6.1 (4.37 ± 1.11)	42	35	Plasma	Cytokines multiplex	RANTES, MIP-1α, MIP-1β	IP-10, MIG	Eotaxin, MCP-1	(71)
2 to 8	2 to 8	36	35	Serum	Cytokines multiplex	IL-8			(72)
(7.4 ± 2.7)	(7.2 ± 1.8)	24	24	Plasma	ELISA, flow cytometry	IFN-γ		IL-1β, TNF-α, IL-10, IL-6, IL-4, IL-2	(73)
(12.21 ± 2.67)	(12.52 ± 2.14)	33	31	Plasma	ELISA	IL-6			(74)

Age range (in years) (mean ± SD; median)		Sample size (N)		Sample type	Analysis techniques	Reported findings in autism compared to controls			Ref.
Autism	Controls	Autism	Controls			↑	↓	≈	
2 to 6 (3)	5 to 16 (10)	30	14	Serum	ELISA	TNF-α, TGF-β, IL-10		IL-1β, IFN-γ, IL-8	(75)
2.2 to 5	2.3 to 4.8	17	16	Plasma	Cytokines multiplex, flow cytometry			IL-12p70, IFN-γ, IL-4, IL-5, IL-10	(6)
3 to 11 (7)	3 to 11 (7)	25	30	Serum	ELISA	IL-1β, IL-18			(18)
3 to 14.5 (6.17 ± 2.58)	3 to 11 (6.75 ± 1.92)	50	30	Serum	Flow cytometry	IL-6, IL-17A		IL-1β, IFN-γ, IL-4	(27)

Symbols: ↑, increased; ↓, decreased; ≈, unchanged; in autism compared to controls.

Abbreviations: 6ckine, ccl21 (chemokine (C-C motif) ligand 21); ATP, adenosine triphosphate; BCA-1, B cell attracting chemokine; BDE-47, tetrabromodiphenylether; CTACK, cutaneous T cell attracting chemokine; ELISA, enzyme linked immunosorbent assay; ENA-78, epithelial neutrophil activating peptide; GCP-2, granulocyte chemotactic protein-2; GM-CSF, granulocyte monocyte colony stimulating factor; GRO, growth-regulated-oncogene; HLA, human leukocyte antigen; I-TAC, interferon inducible T cell alpha chemoattractant; I309, CCL1 (C-C motif chemokine ligand 1); IFN-γ, interferon-γ; Ig, immunoglobulin; IL, interleukin; CD, cluster of differentiation; LPS, lipopolysaccharide; LTA, lipoteichoic acid; MCP-1, monocyte chemoattractant protein-1; MCP, monocyte chemoattractant protein; IP-10, interferon gamma-induced protein; MDC, macrophage derived chemokine; MIF, macrophage migration inhibitory factor; MIG, monokine induced by gamma; MIP, macrophage inflammatory protein; MPIF, myeloid progenitor inhibitory factor; PBMCs, peripheral blood mononuclear cells; PGN, polymeric peptidoglycan; PHA, phytohemagglutinin; PMA, phorbol myristate acetate; poly I:C, polyinosinic:polycytidylic acid; qPCR, quantitative polymerase chain reaction; RANTES, regulated upon activation, normal T cell expressed and presumably secreted; SCYB16, CXCL16 (chemokine C-X-C motif ligand 16); SDF, stromal cell derived factor; TARC, thymus- and activation-regulated chemokine; TARC, thymus- and activation-regulated chemokine; TECK, thymus expressed chemokine; TGF-β, transforming growth factor-β; TNF-α, tumor necrosis factor-α.

References

1. Atwan H, Assarehzadegan MA, Shekarabi M, Jazayeri SM, Barfi S, Shokouhi Shoormasti R, et al. Assessment of miR-181b-5p, miR-23a-3p, BCL-2, and IL-6 in Peripheral Blood Mononuclear Cells of Autistic Patients; Likelihood of Reliable Biomarkers. Iran J Allergy Asthma Immunol. 2020;19(1):74-83.
2. Onore C, Enstrom A, Krakowiak P, Hertz-Picciotto I, Hansen R, Van de Water J, et al. Decreased cellular IL-23 but not IL-17 production in children with autism spectrum disorders. J Neuroimmunol. 2009;216(1-2):126-9.
3. Akintunde ME, Rose M, Krakowiak P, Heuer L, Ashwood P, Hansen R, et al. Increased production of IL-17 in children with autism spectrum disorders and co-morbid asthma. J Neuroimmunol. 2015;286:33-41.
4. Molloy CA, Morrow AL, Meinzen-Derr J, Schleifer K, Dienger K, Manning-Courtney P, et al. Elevated cytokine levels in children with autism spectrum disorder. J Neuroimmunol. 2006;172(1-2):198-205.
5. Ashwood P, Krakowiak P, Hertz-Picciotto I, Hansen R, Pessah IN, Van de Water J. Altered T cell responses in children with autism. Brain Behav Immun. 2011;25(5):840-9.
6. Enstrom AM, Onore CE, Van de Water JA, Ashwood P. Differential monocyte responses to TLR ligands in children with autism spectrum disorders. Brain, behavior, and immunity. 2010;24(1):64-71.
7. Ahmad SF, Ansari MA, Nadeem A, Bakheet SA, Al-Ayadhi LY, Alasmari AF, et al. Involvement of CD45 cells in the development of autism spectrum disorder through dysregulation of granulocyte-macrophage colony-stimulating factor, key inflammatory cytokines, and transcription factors. Int Immunopharmacol. 2020;83:106466.
8. Makinodan M, Iwata K, Ikawa D, Yamashita Y, Yamamuro K, Toritsuka M, et al. Tumor necrosis factor-alpha expression in peripheral blood mononuclear cells correlates with early childhood social interaction in autism spectrum disorder. Neurochem Int. 2017;104:1-5.
9. Ahmad SF, Ansari MA, Ahmed N, Bakheet SA, Al-Ayadhi LY, Attia SM. Elevated IL-16 expression is associated with development of immune dysfunction in children with autism. Psychopharmacology. 2019;236(2):831-8.
10. Ahmad SF, Nadeem A, Ansari MA, Bakheet SA, Attia SM, Zoheir KMA, et al. Imbalance between the anti- and pro-inflammatory milieu in blood leukocytes of autistic children. Molecular Immunology. 2017;82:57-65.
11. Ashwood P, Schauer J, Pessah IN, Van de Water J. Preliminary evidence of the in vitro effects of BDE-47 on innate immune responses in children with autism spectrum disorders. J Neuroimmunol. 2009;208(1-2):130-5.
12. Balestrieri E, Cipriani C, Matteucci C, Benvenuto A, Coniglio A, Argaw-Denboba A, et al. Children With Autism Spectrum Disorder and Their Mothers Share Abnormal Expression of Selected Endogenous Retroviruses Families and Cytokines. Front Immunol. 2019;10:2244.
13. Kutlu A, Cevher Bınıcı N. Does increased neutrophil-lymphocyte ratio predict autism spectrum disorder? Anadolu Psikiyatri Dergisi. 2018;19(6):607-14.
14. Nadeem A, Ahmad SF, Al-Ayadhi LY, Attia SM, Al-Harbi NO, Alzahrani KS, et al. Differential regulation of Nrf2 is linked to elevated inflammation and nitrative stress in monocytes of children with autism. Psychoneuroendocrinology. 2020;113.

15. Nadeem A, Ahmad SF, Al-Harbi NO, Alasmari AF, Al-Ayadhi LY, Alasmari F, et al. Upregulation of enzymatic antioxidants in CD4+ T cells of autistic children. Biochimie. 2020;171-172:205-12.
16. Nadeem A, Ahmad SF, Attia SM, Al-Ayadhi LY, Al-Harbi NO, Bakheet SA. Dysregulation in IL-6 receptors is associated with upregulated IL-17A related signaling in CD4+ T cells of children with autism. Progress in Neuro-Psychopharmacology and Biological Psychiatry. 2020;97.
17. Rose DR, Yang H, Serena G, Sturgeon C, Ma B, Careaga M, et al. Differential immune responses and microbiota profiles in children with autism spectrum disorders and co-morbid gastrointestinal symptoms. Brain, Behavior, and Immunity. 2018;70:354-68.
18. Saresella M, Piancone F, Marventano I, Zoppis M, Hernis A, Zanette M, et al. Multiple inflammasome complexes are activated in autistic spectrum disorders. Brain, Behavior, and Immunity. 2016;57:125-33.
19. Tural Hesapcioglu S, Kasak M, Cıtak Kurt AN, Ceylan MF. High monocyte level and low lymphocyte to monocyte ratio in autism spectrum disorders. International Journal of Developmental Disabilities. 2019;65(2):73-81.
20. Ashwood P, Corbett BA, Kantor A, Schulman H, Van de Water J, Amaral DG. In search of cellular immunophenotypes in the blood of children with autism. PLoS One. 2011;6(5):e19299.
21. Mostafa GA, Al Shehab A, Fouad NR. Frequency of CD4+CD25high regulatory T cells in the peripheral blood of Egyptian children with autism. J Child Neurol. 2010;25(3):328-35.
22. Siniscalco D, Mijatovic T, Bosmans E, Cirillo A, Kruzliak P, Lombardi VC, et al. Decreased Numbers of CD57+CD3- Cells Identify Potential Innate Immune Differences in Patients with Autism Spectrum Disorder. In Vivo. 2016;30(2):83-9.
23. Wasilewska J, Kaczmarski M, Stasiak-Barmuta A, Tobolczyk J, Kowalewska E. Low serum IgA and increased expression of CD23 on B lymphocytes in peripheral blood in children with regressive autism aged 3-6 years old. Arch Med Sci. 2012;8(2):324-31.
24. Breece E, Paciotti B, Nordahl CW, Ozonoff S, Van de Water JA, Rogers SJ, et al. Myeloid dendritic cells frequencies are increased in children with autism spectrum disorder and associated with amygdala volume and repetitive behaviors. Brain Behav Immun. 2013;31:69-75.
25. Enstrom AM, Lit L, Onore CE, Gregg JP, Hansen RL, Pessah IN, et al. Altered gene expression and function of peripheral blood natural killer cells in children with autism. Brain Behav Immun. 2009;23(1):124-33.
26. Ashwood P. Differential T Cell Levels of Tumor Necrosis Factor Receptor-II in Children With Autism. Front Psychiatry. 2018;9:543.
27. Basheer S, Venkataswamy MM, Christopher R, Van Amelsvoort T, Srinath S, Girimaji SC, et al. Immune aberrations in children with Autism Spectrum Disorder: a case-control study from a tertiary care neuropsychiatric hospital in India. Psychoneuroendocrinology. 2018;94:162-7.
28. Bennabi M, Tarantino N, Gaman A, Scheid I, Krishnamoorthy R, Debre P, et al. Persistence of dysfunctional natural killer cells in adults with high-functioning autism spectrum disorders: stigma/consequence of unresolved early infectious events? Mol Autism. 2019;10:22.
29. Heuer LS, Rose M, Ashwood P, Van de Water J. Decreased levels of total immunoglobulin in children with autism are not a result of B cell dysfunction. J Neuroimmunol. 2012;251(1-2):94-102.
30. Abruzzo PM, Matte A, Bolotta A, Federti E, Ghezzo A, Guarnieri T, et al. Plasma peroxiredoxin changes and inflammatory cytokines support the involvement of neuro-inflammation and oxidative stress in Autism Spectrum Disorder. J Transl Med. 2019;17(1):332.

31. Ahmad SF, Nadeem A, Ansari MA, Bakheet SA, Al-Ayadhi LY, Attia SM. Upregulation of IL-9 and JAK-STAT signaling pathway in children with autism. Prog Neuropsychopharmacol Biol Psychiatry. 2017;79(Pt B):472-80.
32. Nadeem A, Ahmad SF, Attia SM, Al-Ayadhi LY, Bakheet SA, Al-Harbi NO. Oxidative and inflammatory mediators are upregulated in neutrophils of autistic children: Role of IL-17A receptor signaling. Prog Neuropsychopharmacol Biol Psychiatry. 2019;90:204-11.
33. Kutuk MO, Tufan E, Gokcen C, Kilicaslan F, Karadag M, Mutluer T, et al. Cytokine expression profiles in Autism spectrum disorder: A multi-center study from Turkey. Cytokine. 2020;133.
34. Ahmad SF, Ansari MA, Nadeem A, Bakheet SA, Al-Ayadhi LY, Alotaibi MR, et al. Dysregulation of T cell immunoglobulin and mucin domain 3 (TIM-3) signaling in peripheral immune cells is associated with immune dysfunction in autistic children. Molecular Immunology. 2019;106:77-86.
35. Eftekharian MM, Ghafouri-Fard S, Noroozi R, Omrani MD, Arsang-jang S, Ganji M, et al. Cytokine profile in autistic patients. Cytokine. 2018;108:120-6.
36. Okada K, Hashimoto K, Iwata Y, Nakamura K, Tsujii M, Tsuchiya KJ, et al. Decreased serum levels of transforming growth factor-beta1 in patients with autism. Progress in neuro-psychopharmacology & biological psychiatry. 2007;31(1):187-90.
37. Ashwood P, Enstrom A, Krakowiak P, Hertz-Picciotto I, Hansen RL, Croen LA, et al. Decreased transforming growth factor beta1 in autism: a potential link between immune dysregulation and impairment in clinical behavioral outcomes. Journal of neuroimmunology. 2008;204(1-2):149-53.
38. Al-ayadhi LY, Mostafa GA. Increased serum osteopontin levels in autistic children: relation to the disease severity. Brain Behav Immun. 2011;25(7):1393-8.
39. Ashwood P, Krakowiak P, Hertz-Picciotto I, Hansen R, Pessah IN, Van de Water J. Associations of impaired behaviors with elevated plasma chemokines in autism spectrum disorders. Journal of neuroimmunology. 2011;232(1-2):196-9.
40. Manzardo AM, Henkhaus R, Dhillon S, Butler MG. Plasma cytokine levels in children with autistic disorder and unrelated siblings. International journal of developmental neuroscience : the official journal of the International Society for Developmental Neuroscience. 2012;30(2):121-7.
41. Mostafa GA, Al-Ayadhi LY. The possible link between elevated serum levels of epithelial cell-derived neutrophil-activating peptide-78 (ENA-78/CXCL5) and autoimmunity in autistic children. Behav Brain Funct. 2015;11:11-.
42. Al-Ayadhi LY, Mostafa GA. Elevated serum levels of macrophage-derived chemokine and thymus and activation-regulated chemokine in autistic children. Journal of neuroinflammation. 2013;10:72-.
43. Ashwood P, Krakowiak P, Hertz-Picciotto I, Hansen R, Pessah I, Van de Water J. Elevated plasma cytokines in autism spectrum disorders provide evidence of immune dysfunction and are associated with impaired behavioral outcome. Brain, behavior, and immunity. 2011;25(1):40-5.
44. Krakowiak P, Goines PE, Tancredi DJ, Ashwood P, Hansen RL, Hertz-Picciotto I, et al. Neonatal Cytokine Profiles Associated With Autism Spectrum Disorder. Biol Psychiatry. 2017;81(5):442-51.
45. Zerbo O, Yoshida C, Grether JK, Van de Water J, Ashwood P, Delorenze GN, et al. Neonatal cytokines and chemokines and risk of Autism Spectrum Disorder: the Early Markers for Autism (EMA) study: a case-control study. Journal of neuroinflammation. 2014;11:113.
46. Abdallah MW, Mortensen EL, Greaves-Lord K, Larsen N, Bonefeld-Jørgensen EC, Nørgaard-Pedersen B, et al. Neonatal levels of neurotrophic factors and risk of autism spectrum disorders. Acta Psychiatr Scand. 2013;128(1):61-9.

47. Abdallah MW, Larsen N, Grove J, Bonefeld-Jorgensen EC, Norgaard-Pedersen B, Hougaard DM, et al. Neonatal chemokine levels and risk of autism spectrum disorders: findings from a Danish historic birth cohort follow-up study. Cytokine. 2013;61(2):370-6.
48. Abdallah MW, Larsen N, Mortensen EL, Atladottir HO, Norgaard-Pedersen B, Bonefeld-Jorgensen EC, et al. Neonatal levels of cytokines and risk of autism spectrum disorders: an exploratory register-based historic birth cohort study utilizing the Danish Newborn Screening Biobank. Journal of neuroimmunology. 2012;252(1-2):75-82.
49. Xie J, Huang L, Li X, Li H, Zhou Y, Zhu H, et al. Immunological cytokine profiling identifies TNF-α as a key molecule dysregulated in autistic children. Oncotarget. 2017;8(47):82390-8.
50. Ghaffari MA, Mousavinejad E, Riahi F, Mousavinejad M, Afsharmanesh MR. Increased Serum Levels of Tumor Necrosis Factor-Alpha, Resistin, and Visfatin in the Children with Autism Spectrum Disorders: A Case-Control Study. Neurol Res Int. 2016;2016:9060751-.
51. Heuer LS, Croen LA, Jones KL, Yoshida CK, Hansen RL, Yolken R, et al. An Exploratory Examination of Neonatal Cytokines and Chemokines as Predictors of Autism Risk: The Early Markers for Autism Study. Biological psychiatry. 2019;86(4):255-64.
52. Abd-Allah NA, Ibrahim OM, Elmalt HA, Shehata MA, Hamed RA, Elsaadouni NM, et al. Thioredoxin level and inflammatory markers in children with autism spectrum disorders. Middle East Current Psychiatry. 2020;27(1).
53. Al-Ayadhi LY, Mostafa GA. Elevated serum levels of interleukin-17A in children with autism. J Neuroinflammation. 2012;9:158.
54. Barbosa IG, Rodrigues DH, Rocha NP, Sousa LFDC, Vieira ELM, Simões-e-Silva AC, et al. Plasma levels of alarmin IL-33 are unchanged in autism spectrum disorder: A preliminary study. Journal of Neuroimmunology. 2015;278:69-72.
55. Hu CC, Xu X, Xiong GL, Xu Q, Zhou BR, Li CY, et al. Alterations in plasma cytokine levels in chinese children with autism spectrum disorder. Autism Research. 2018;11(7):989-99.
56. El Gohary TM, El Aziz NA, Darweesh M, Sadaa ES. Plasma level of transforming growth factor β 1 in children with autism spectrum disorder. Egyptian Journal of Ear, Nose, Throat and Allied Sciences. 2015;16(1):69-73.
57. El Wakkad ASED, Saleh MT. The proinflammatory cytokines in children with autism. Pakistan Journal of Biological Sciences. 2006;9(14):2593-9.
58. El-Ansary A, Al-Ayadhi L. Neuroinflammation in autism spectrum disorders. Journal of Neuroinflammation. 2012;9:265.
59. El-Ansary A, Al-Ayadhi L. GABAergic/glutamatergic imbalance relative to excessive neuroinflammation in autism spectrum disorders. J Neuroinflammation. 2014;11:189.
60. Enstrom A, Onore C, Hertz-Picciotto I, Hansen R, Croen L, Van De Water J, et al. Detection of IL-17 and IL-23 in plasma samples of children with autism. American Journal of Biochemistry and Biotechnology. 2008;4(2):114-20.
61. Guloksuz SA, Abali O, Aktas Cetin E, Bilgic Gazioglu S, Deniz G, Yildirim A, et al. Elevated plasma concentrations of S100 calcium-binding protein B and tumor necrosis factor alpha in children with autism spectrum disorders. Braz J Psychiatry. 2017;39(3):195-200.
62. Hamed NO, Laila Al A, Osman MA, Elkhawad AO, Bjørklund G, Qasem H, et al. Determination of neuroinflammatory biomarkers in autistic and neurotypical Saudi children. Metabolic Brain Disease. 2019;34(4):1049-60.

63. Hashim H, Abdelrahman H, Mohammed D, Karam R. Association between plasma levels of transforming growth factor-β1, IL-23 and IL-17 and the severity of autism in Egyptian children. Research in Autism Spectrum Disorders. 2013;7(1):199-204.
64. Kordulewska NK, Kostyra E, Piskorz-Ogórek K, Moszyńska M, Cieślińska A, Fiedorowicz E, et al. Serum cytokine levels in children with spectrum autism disorder: Differences in pro- and anti-inflammatory balance. Journal of Neuroimmunology. 2019;337.
65. Napolioni V, Ober-Reynolds B, Szelinger S, Corneveaux JJ, Pawlowski T, Ober-Reynolds S, et al. Plasma cytokine profiling in sibling pairs discordant for autism spectrum disorder. Journal of Neuroinflammation. 2013;10:38.
66. Ning J, Xu L, Shen CQ, Zhang YY, Zhao Q. Increased serum levels of macrophage migration inhibitory factor in autism spectrum disorders. NeuroToxicology. 2019;71:1-5.
67. Ricci S, Businaro R, Ippoliti F, Lo Vasco VR, Massoni F, Onofri E, et al. Altered cytokine and BDNF levels in autism spectrum disorder. Neurotoxicity Research. 2013;24(4):491-501.
68. Rose D, Ashwood P. Rapid communication: Plasma interleukin-35 in children with Autism. Brain Sciences. 2019;9(7).
69. Saad K, Abdallah AM, Abdel-Rahman AA, Al-Atram AA, Abdel-Raheem YF, Gad EF, et al. Polymorphism of interleukin-1β and interleukin-1 receptor antagonist genes in children with autism spectrum disorders. Prog Neuropsychopharmacol Biol Psychiatry. 2020;103:109999.
70. Shen Y, Li Y, Shi L, Liu M, Wu R, Xia K, et al. Autism spectrum disorder and severe social impairment associated with elevated plasma interleukin-8. Pediatr Res. 2020.
71. Shen Y, Oua J, Liu M, Shi L, Li Y, Xiao L, et al. Altered plasma levels of chemokines in autism and their association with social behaviors. Psychiatry Research. 2016;244:300-5.
72. Singh S, Yazdani U, Gadad B, Zaman S, Hynan LS, Roatch N, et al. Serum thyroid-stimulating hormone and interleukin-8 levels in boys with autism spectrum disorder. Journal of Neuroinflammation. 2017;14.
73. Tostes MHFS, Teixeira HC, Gattaz WF, Brandão MAF, Raposo NRB. Altered neurotrophin, neuropeptide, cytokines and nitric oxide levels in autism. Pharmacopsychiatry. 2012;45(6):241-3.
74. Yang CJ, Liu CL, Sang B, Zhu XM, Du YJ. The combined role of serotonin and interleukin-6 as biomarker for autism. Neuroscience. 2015;284:290-6.
75. Carissimi C, Laudadio I, Palone F, Fulci V, Cesi V, Cardona F, et al. Functional analysis of gut microbiota and immunoinflammation in children with autism spectrum disorders. Dig Liver Dis. 2019;51(10):1366-74.
