# Supplementary material for: In search of immune cellular sources of abnormal cytokines in the blood in autism spectrum disorder: A systematic review of case-control studies
Source: Front Immunol. 2022 Oct 4;13:950275. doi: 10.3389/fimmu.2022.950275 (PMC9578337; doi:10.3389/fimmu.2022.950275)
Supplement: Supplementary file 1 [file DataSheet_1.zip › Supplementary material/Table S5.pdf]

Table S5. Quality assessment of the eligible articles followed by inclusion of at least fair quality rated articles.

Study	C1	C2	C3	C4	C5	C6	C7	C8	C9	C10	C11	C12	Rating
(1) Abd-Allah, N. A., et al., 2020	YES	YES	NR	NO	NR	YES	NA	NA	NA	YES	NR	NA	Fair
(2) Abdallah, M. W., et al., 2012	YES	YES	NR	YES	NR	NR	NA	YES	No	YES	NR	NA	Fair
(3) Abdallah, M. W., et al., 2013	YES	YES	NR	YES	NR	NR	NA	YES	NA	YES	NR	YES	Fair
(4) Abdallah, M. W., et al., 2013	YES	YES	NR	YES	NR	NR	NA	YES	NA	YES	NR	NA	Fair
(5) Abdel-Salam, O. M. E., et al., 2017	YES	NO	NR	NR	NR	YES	NA	NR	NA	YES	NR	CD	Poor
(6) Abruzzo, P. M., et al., 2019	YES	NO	NR	Yes	NR	NR	NA	NR	NA	YES	NR	YES	Fair
(7) Ahmad, S. F., et al., 2017	YES	YES	NR	NR	NR	YES	NA	NR	NA	YES	NR	NA	Fair
(8) Ahmad, S. F., et al., 2017	YES	YES	NR	NR	NR	YES	NA	NR	NA	YES	NR	NA	Fair
(9) Ahmad, S. F., et al., 2019	YES	YES	NR	NR	NR	YES	NA	NR	NA	YES	NR	CD	Fair
(10) Ahmad, S. F., et al., 2019	YES	YES	NR	NR	NR	YES	NA	NR	NA	YES	NR	NA	Fair
(11) Ahmad, S. F., et al., 2020	YES	YES	NR	NR	NR	YES	NA	NR	NA	YES	NR	NA	Fair
(12) Akintunde, M. E., et al., 2015	YES	YES	NR	NR	YES	NR	NO	NR	NA	YES	NR	YES	Fair
(13) Al-Ayadhi, L. Y., 2005	YES	NO	NR	NR	NR	NR	NA	NR	NA	YES	NR	NA	Poor
(14) Al-ayadhi, L. Y. and G. A. Mostafa, 2011	YES	YES	NR	YES	NR	YES	NA	YES	NA	YES	NR	NA	Fair
(15) Al-Ayadhi, L. Y. and G. A. Mostafa, 2012	YES	YES	NR	NR	NR	YES	NA	NR	NA	YES	NR	NA	Fair
(16) Al-Ayadhi, L. Y. and G. A. Mostafa, 2013	YES	YES	NR	NR	NR	YES	NA	NR	NA	YES	NR	NA	Fair
(17) Alzghoul, L., et al., 2019	YES	NO	NR	NR	NR	YES	NA	NR	NA	YES	NR	NA	Poor
(18) Ashaat, E. A., et al., 2017	YES	YES	NR	NR	NR	NO	NA	NR	NA	NO	NR	NO	Poor
(19) Ashwood, P., 2018	YES	NO	NR	NR	YES	YES	NA	NR	NA	YES	NR	NA	Fair
(20) Ashwood, P., et al., 2008	YES	YES	NR	NR	YES	YES	NA	NR	NA	YES	NR	NA	Fair
(21) Ashwood, P., et al., 2009	YES	YES	NR	NR	YES	YES	NA	NR	NA	YES	NR	NA	Fair
(22) Ashwood, P., et al., 2011	YES	YES	NR	NR	YES	YES	NO	NR	NA	YES	NR	NA	Fair
(23) Ashwood, P., et al., 2011	YES	YES	NR	NR	YES	YES	NA	NR	NA	YES	NR	NA	Fair
(24) Ashwood, P., et al., 2011	YES	YES	NR	NR	YES	YES	NA	NR	NA	YES	NR	NA	Fair
(25) Ashwood, P., et al., 2011	YES	YES	NR	NR	YES	YES	NA	NR	NA	YES	NR	NA	Fair

Study	C1	C2	C3	C4	C5	C6	C7	C8	C9	C10	C11	C12	Rating
(26) Atwan, H., et al., 2020	YES	YES	NR	NR	NR	YES	NA	NR	NA	YES	NR	NA	Fair
(27) Balestrieri, E., et al., 2019	YES	YES	NR	NR	NR	YES	NA	NR	NA	YES	NR	NA	Fair
(28) Barbosa, I. G., et al., 2015	YES	YES	NR	YES	NR	YES	NA	NR	NA	YES	NR	NA	Fair
(29) Basheer, S., et al., 2018	YES	YES	NR	YES	YES	YES	NA	NR	NA	YES	NR	NA	Fair
(30) Bennabi, M., et al., 2019	YES	YES	NR	NR	NR	YES	NA	NR	NA	YES	NR	NA	Fair
(31) Bijl, N., et al., 2015	YES	YES	NR	NR	NR	NR	NA	NR	NA	YES	NR	No	Poor
(32) Breece, E., et al., 2013	YES	YES	NR	NR	YES	YES	NA	NR	NA	YES	NR	NA	Fair
(33) Bressler, J.P., et al., 2012	YES	YES	NR	NR	NR	NR	NA	NR	NA	YES	NR	NA	Poor
(34) Bryn, V., et al., 2017	YES	YES	NR	NR	NR	NR	NA	NR	NA	YES	NR	NA	Poor
(35) Businaro, R., et al., 2016	YES	YES	NR	NR	NR	NR	NA	NR	NA	NR	NR	NO	Poor
(36) Carissimi, C., et al., 2019	YES	YES	NR	NR	NR	YES	NA	NR	NA	YES	NR	NA	Fair
(37) Chun-Chun, H., et al., 2018	YES	YES	NR	YES	NR	YES	NA	NR	NA	YES	NR	NA	Fair
(38) Croonenberghs, J., et al., 2002	YES	NO	NR	NR	NR	YES	NA	NR	NA	YES	NR	NA	Poor
(39) Denney, D. R., et al., 1996	YES	YES	NR	NR	NR	NR	NA	NR	NA	YES	NR	NA	Poor
(40) Eftekharian, M. M., et al., 2018	YES	YES	NR	NR	NR	YES	NA	NR	NA	YES	NR	NA	Fair
(41) El Gohary, T. M., et al., 2015	YES	NO	NR	NR	YES	YES	NA	NR	NA	YES	NR	NA	Fair
(42) El Wakkad, A. S. E. D. and M. T. Saleh, 2006	YES	NO	NR	NR	YES	YES	NA	NR	NA	YES	NR	NA	Fair
(43) El-Ansary, A. and L. Al-Ayadhi, 2012	YES	YES	NR	NR	NR	YES	NA	NR	NA	YES	NR	NA	Fair
(44) El-Ansary, A. and L. Al-Ayadhi, 2014	YES	YES	NR	NR	NR	YES	NA	NR	NA	YES	NR	NA	Fair
(45) El-Ansary, A. K., et al., 2011	YES	YES	NR	NR	NR	YES	NA	NR	NA	NO	NR	NA	Poor
(46) El-Ansary, A., et al., 2016	YES	YES	NR	NR	NR	YES	NA	NR	NA	NO	NR	NA	Poor
(47) Emanuele, E., et al., 2010	YES	YES	NR	NR	NR	NR	NA	NR	NA	YES	NR	NA	Poor
(48) Engstrom, H. A., et al., 2003	YES	NO	NR	NR	NR	NR	NA	NR	NA	YES	NR	NA	Poor
(49) Enstrom, A. M., et al., 2009	YES	YES	NR	NR	YES	NR	NA	NR	NA	YES	NR	NA	Fair
(50) Enstrom, A. M., et al., 2010	YES	YES	NR	NR	YES	NR	NA	NR	NA	YES	NR	NA	Fair
(51) Enstrom, A., et al., 2008	YES	YES	NR	NR	YES	NR	NA	NR	NA	YES	NR	NA	Fair
(52) Esnafoglu, E. and S. N. Ayyıldız, 2017	YES	YES	NR	NR	NR	NR	NA	NR	NA	YES	NR	NA	Poor

Study	C1	C2	C3	C4	C5	C6	C7	C8	C9	C10	C11	C12	Rating
(53) Ferrante, P., et al., 2003	YES	NO	NR	NR	NR	NR	NA	NR	NA	CD	NR	NA	Poor
(54) Ghaffari, M. A., et al., 2016	YES	Yes	NR	YES	NR	NR	NA	NR	NA	YES	NR	NA	Fair
(55) Gomez-Fernandez, A., et al., 2018	YES	NO	NR	NR	NR	YES	NA	NR	NA	YES	NR	NA	Poor
(56) Guloksuz, S. A., et al., 2017	YES	YES	NR	YES	NR	YES	NA	NR	NA	YES	NR	NA	Fair
(57) Gupta, S., et al., 1998	YES	NO	NR	NR	NR	NR	NA	NR	NA	YES	NR	NA	Poor
(58) Hamed, N. O., et al., 2019	YES	YES	NR	NO	NR	YES	NA	NR	NA	YES	NR	NA	Fair
(59) Han, Y. M., et al., 2017	YES	YES	NR	NR	NR	NR	NA	NR	NA	YES	NR	NA	Poor
(60) Hashim, H., et al., 2013	YES	YES	NR	NR	YES	YES	NA	NR	NA	YES	NR	NA	Fair
(61) Heuer, L. S., et al., 2012	YES	YES	NR	YES	YES	Yes	NA	NR	NA	YES	NR	NA	Fair
(62) Heuer, L. S., et al., 2019	YES	YES	NR	YES	YES	NR	NA	NR	YES	YES	NR	NA	Fair
(63) Hollander, E., et al., 1999	YES	NO	NR	NR	NR	NR	NA	NR	NA	YES	NR	NA	Poor
(64) Hunter, L. C., et al., 2003	YES	YES	NR	NR	NR	NR	NA	NR	NA	YES	NR	NA	Poor
(65) Inga Jácome, M. C., et al., 2016	YES	NO	NR	NR	NR	NR	NA	NR	NA	YES	NR	NA	Poor
(66) Jyonouchi, H., et al., 2001	YES	NO	NR	NR	NR	NR	NA	NR	NA	YES	NR	NA	Poor
(67) Jyonouchi, H., et al., 2002	YES	NO	NR	NR	NR	NR	NA	NR	NA	YES	NR	NA	Poor
(68) Jyonouchi, H., et al., 2005	YES	NO	NR	NR	NR	YES	NA	NR	NA	YES	NR	NA	Poor
(69) Jyonouchi, H., et al., 2005	YES	NO	NR	NR	NR	YES	NA	NR	NA	YES	NR	NA	Poor
(70) Jyonouchi, H., et al., 2008	YES	YES	NR	NR	NR	NR	NA	NR	NA	YES	NR	NA	Poor
(71) Jyonouchi, H., et al., 2011	YES	YES	NR	NR	NR	NR	NA	NR	NA	YES	NR	NA	Poor
(72) Jyonouchi, H., et al., 2012	YES	YES	NR	NR	NR	NR	NA	NR	NA	YES	NR	NA	Poor
(73) Jyonouchi, H., et al., 2014	YES	NO	NR	NR	NR	YES	NA	NR	NA	YES	NR	NA	Poor
(74) Jyonouchi, H., et al., 2017	YES	YES	NR	NR	NR	NR	NA	NR	NA	YES	NR	NA	Poor
(75) Jyonouchi, H., et al., 2019	YES	NO	NR	NR	NR	YES	NA	NR	NA	YES	NR	NA	Poor
(76) Kordulewska, N. K., et al., 2019	YES	YES	NR	NR	YES	Yes	NA	NR	NA	YES	NR	NA	Fair
(77) Krakowiak, P., et al., 2017	YES	YES	NR	YES	YES	YES	NA	NR	NA	YES	NR	NA	Fair
(78) Kutlu, A. and N. Cevher Bınıcı, 2018	YES	YES	NR	NR	YES	YES	NA	YES	NA	YES	NR	NA	Fair
(79) Kutuk, M. O., et al., 2020	YES	YES	NR	NR	YES	YES	NA	NR	NA	YES	NR	NA	Fair

Study	C1	C2	C3	C4	C5	C6	C7	C8	C9	C10	C11	C12	Rating
(80) Lochman, I., et al., 2018	YES	YES	NR	YES	NO	NR	NO	NR	NA	NO	NR	NA	Poor
(81) López-Cacho, J. M., et al., 2016	YES	YES	NR	NR	NR	NR	NO	NR	NA	YES	NR	NA	Poor
(82) Magid-Bernstein, J., et al., 2015	YES	NR	NR	NR	NR	NR	NA	NR	NA	YES	NR	NA	Poor
(83) Makinodan, M., et al., 2017	YES	YES	NR	NR	NR	Yes	NA	NR	NA	YES	NR	NA	Fair
(84) Malik, M., et al., 2011	YES	NO	NR	NR	CD	NR	NA	NR	NA	YES	NR	NA	Poor
(85) Manzardo, A. M., et al., 2012	YES	YES	NR	NR	YES	YES	NA	NR	NA	YES	NR	NA	Fair
(86) Mills, J. L., et al., 2007	YES	YES	NR	NR	NR	NR	NO	NR	NA	YES	NR	NA	Poor
(87) Mizejewski, G. J., et al., 2013	YES	YES	NR	NR	NR	NR	NO	CD	Yes	NO	NR	NA	Poor
(88) Moaaz, M., et al., 2019	YES	YES	NR	NR	NR	NR	NA	NR	NA	YES	NR	NA	Poor
(89) Molloy, C. A., et al., 2006	YES	YES	NR	NR	YES	YES	NA	NR	NA	YES	NR	NA	Fair
(90) Mostafa, G. A. and L. Y. Al-Ayadhi, 2015	YES	YES	NR	NR	NR	YES	NA	NR	NA	YES	NR	NA	Fair
(91) Mostafa, G. A., et al., 2010	YES	YES	NR	YES	NR	YES	NA	NR	NA	YES	NR	NA	Fair
(92) Nadeem, A., et al., 2019	YES	YES	NR	NR	NR	YES	NA	NR	NA	YES	NR	NA	Fair
(93) Nadeem, A., et al., 2020	YES	YES	NR	NR	NR	YES	NA	NR	NA	YES	NR	NA	Fair
(94) Nadeem, A., et al., 2020	YES	YES	NR	NR	NR	YES	NA	NR	NA	YES	NR	NA	Fair
(95) Nadeem, A., et al., 2020	YES	YES	NR	NR	NR	YES	NA	NR	NA	YES	NR	NA	Fair
(96) Napolioni, V., et al., 2013	YES	YES	NR	NR	YES	NR	NA	NR	NA	YES	NR	NA	Fair
(97) Nelson, P. G., et al., 2006	YES	YES	NR	NR	NR	NR	NA	NR	NA	YES	NR	NA	Poor
(98) Ning, J., et al., 2019	YES	YES	NR	YES	YES	NR	NA	NR	NA	YES	NR	NA	Fair
(99) Okada, K., et al., 2007	YES	YES	NR	NR	NO	YES	NA	NR	NA	YES	NR	NA	Fair
(100) Onore, C., et al., 2009	YES	YES	NR	NR	YES	YES	NA	NR	NA	YES	NR	NA	Fair
(101) Pardo, C. A., et al., 2017	YES	YES	NR	NR	NR	NR	NA	NR	NA	YES	NR	NA	Poor
(102) Pecorelli, A., et al., 2016	YES	YES	NR	NR	NR	NR	NA	NR	NA	YES	NR	NA	Poor
(103) Plioplys, A. V., et al., 1994	YES	NO	NR	NR	NR	NR	NA	NR	NA	YES	NR	NA	Poor
(104) Ramsey, J. M., et al., 2013	YES	YES	NR	NR	NR	NR	NA	NR	NA	YES	NR	NA	Poor
(105) Ricci, S., et al., 2013	YES	YES	NR	NR	NR	YES	NA	NR	NA	YES	NR	NA	Fair
(106) Rodrigues, D. H., et al., 2014	YES	YES	NR	NR	NR	NR	NA	NR	NA	YES	NR	NA	Poor

Study	C1	C2	C3	C4	C5	C6	C7	C8	C9	C10	C11	C12	Rating
(107) Rose, D. and P. Ashwood, 2019	YES	YES	NR	Yes	YES	NR	NO	NR	NA	YES	NR	NA	Fair
(108) Rose, D. R., et al., 2018	YES	YES	NR	Yes	YES	NR	NO	NR	NA	YES	NR	NA	Fair
(109) Russo, A. J., 2013	YES	NO	NR	YES	NR	NR	NA	NR	NA	YES	NR	NA	Poor
(110) Russo, A. J., 2014	YES	NO	NR	YES	NR	NR	NA	NR	NA	YES	NR	NA	Poor
(111) Russo, A. J., 2015	YES	NO	NR	YES	NR	NR	NA	NR	NA	YES	NR	NA	Poor
(112) Russo, A. J., et al., 2009	YES	YES	NR	NR	NR	NR	NA	NR	NA	YES	NR	NA	Poor
(113) Saad, K., et al., 2017	YES	YES	NR	NR	NR	NR	NO	NR	NA	YES	NR	NA	Poor
(114) Saad, K., et al., 2020	YES	YES	YES	NR	NR	YES	NA	NR	NA	YES	NR	NA	Fair
(115) Saresella, M., et al., 2009	YES	NO	NR	NR	NR	YES	NA	NR	NA	YES	NR	NA	Poor
(116) Saresella, M., et al., 2016	YES	YES	NR	NR	NR	YES	NA	NR	NA	YES	NR	NA	Fair
(117) Shen, L., et al., 2019	YES	YES	NR	NR	NR	NR	NA	NR	NA	YES	NR	NA	Poor
(118) Shen, Y., et al., 2016	YES	YES	NR	NR	NR	YES	NA	NR	NA	YES	NR	NA	Fair
(119) Shen, Y., et al., 2020	YES	YES	NR	NR	NR	YES	NA	NR	NA	YES	NR	NA	Fair
(120) Singh, S., et al., 2017	YES	YES	NR	NR	NR	YES	NA	NR	NA	YES	NR	NA	Fair
(121) Singh, V. K., 1996	YES	NO	NR	NR	NR	NR	NA	NR	NA	YES	NR	NA	Poor
(122) Singh, V. K., et al., 1988	YES	NO	NR	NR	NR	NR	NA	NR	NA	YES	NR	NA	Poor
(123) Singh, V. K., et al., 1991	YES	Yes	NR	NR	NR	NR	NA	NR	NA	YES	NR	NA	Poor
(124) Siniscalco, D., et al., 2016	YES	YES	NR	NR	YES	NR	NA	NR	NA	YES	NR	NA	Fair
(125) Stubbs, E. G. and M. L. Crawford, 1977	YES	YES	NR	NR	NR	NR	NA	NR	NA	YES	NR	NA	Poor
(126) Suzuki, K., et al., 2007	YES	YES	NR	NR	NO	NR	NA	NR	NA	YES	NR	NA	Poor
(127) Suzuki, K., et al., 2011	YES	NO	NR	NR	NR	NR	NA	NR	NA	YES	NR	NA	Poor
(128) Sweeten, T. L., et al., 2003	YES	YES	NR	NR	NR	NR	NA	NR	NA	YES	NR	NA	Poor
(129) Sweeten, T. L., et al., 2004	YES	YES	NR	NR	NR	NR	NA	NR	NA	YES	NR	NA	Poor
(130) Tobiasova, Z., et al., 2011	YES	YES	NR	NR	NR	NR	NO	NR	NA	YES	NR	NA	Poor
(131) Tonhajzerova, I., et al., 2015	YES	YES	NR	NR	NR	NR	NA	NR	NA	YES	NR	NA	Poor
(132) Tostes, M. H., et al., 2012	YES	YES	NR	NR	NR	YES	NA	NR	NA	YES	NR	NA	Fair
(133) Tsilioni, I. and T. C. Theoharides, 2018	YES	YES	NR	NR	NR	NR	NA	NR	NA	YES	NR	NA	Poor

Study	C1	C2	C3	C4	C5	C6	C7	C8	C9	C10	C11	C12	Rating
(134) Tsilioni, I., et al., 2015	YES	YES	NR	NR	NR	NR	NA	NR	NA	YES	NR	NA	Poor
(135) Tural Hesapcioglu, S., et al., 2019	YES	YES	NR	YES	NR	NR	NA	NR	NA	YES	NR	NA	Fair
(136) Vojdani, A., et al., 2008	YES	YES	NR	NR	NR	NR	NA	NR	NA	YES	NR	NA	Poor
(137) Warren, R. P., et al., 1986	YES	NO	NR	NR	NR	NR	NA	NR	NA	YES	NR	NA	Poor
(138) Warren, R. P., et al., 1987	YES	NO	NR	NR	NR	NR	NA	NR	NA	YES	NR	NA	Poor
(139) Warren, R. P., et al., 1990	YES	NO	NR	NR	NR	NR	NA	NR	NA	YES	NR	NA	Poor
(140) Warren, R. P., et al., 1995	YES	NO	NR	NR	NR	NR	NA	NR	NA	YES	NR	NA	Poor
(141) Wasilewska, J., et al., 2012	YES	YES	NR	YES	NO	YES	NA	NR	NA	YES	NR	NA	Fair
(142) Xie, J., et al., 2017	YES	YES	NR	NR	YES	NR	NA	NR	NA	YES	NR	NA	Fair
(143) Yang, C. J., et al., 2015	YES	YES	NR	NR	NR	YES	NA	NR	NA	YES	NR	NA	Fair
(144) Yonk, L. J., et al., 1990	YES	NO	NR	NR	NR	NR	NA	NR	NA	YES	NR	NA	Poor
(145) Zerbo, O., et al., 2014	YES	YES	NR	NR	NR	NR	YES	NR	YES	YES	NR	NA	Fair
(146) Zimmerman, A. W., et al., 2005	YES	YES	NR	NR	NR	NR	NA	NR	NA	YES	NR	NA	Poor

Available at: [https://www.nhlbi.nih.gov/health-topics/study-quality-assessment-tools].
C, criteria; CD, cannot determine; NA, not applicable; NR, not reported.
Rating based on Yes (0-3 Poor); (4-6 Fair); (7-12 Good).

References

1. Abd-Allah NA, Ibrahim OM, Elmalt HA, Shehata MA, Hamed RA, Elsaadouni NM, et al. Thioredoxin level and inflammatory markers in children with autism spectrum disorders. Middle East Current Psychiatry. 2020;27(1).
2. Abdallah MW, Larsen N, Mortensen EL, Atladóttir HÓ, Nørgaard-Pedersen B, Bonefeld-Jørgensen EC, et al. Neonatal levels of cytokines and risk of autism spectrum disorders: An exploratory register-based historic birth cohort study utilizing the Danish Newborn Screening Biobank. Journal of Neuroimmunology. 2012;252(1-2):75-82.
3. Abdallah MW, Larsen N, Grove J, Bonefeld-Jørgensen EC, Nørgaard-Pedersen B, Hougaard DM, et al. Neonatal chemokine levels and risk of autism spectrum disorders: Findings from a Danish historic birth cohort follow-up study. Cytokine. 2013;61(2):370-6.
4. Abdallah MW, Mortensen EL, Greaves-Lord K, Larsen N, Bonefeld-Jørgensen EC, Nørgaard-Pedersen B, et al. Neonatal levels of neurotrophic factors and risk of autism spectrum disorders. Acta Psychiatr Scand. 2013;128(1):61-9.
5. Abdel-Salam OME, Youness ER, Abu Elhamed WA. Changes in monocyte chemoattractive protein, nuclear respiratory factor 2, B-cell leukemia/lymphoma 2 and cholinesterase in serum of autistic children. Biomedical and Pharmacology Journal. 2017;10(2):659-66.
6. Abruzzo PM, Matté A, Bolotta A, Federti E, Ghezzo A, Guarnieri T, et al. Plasma peroxiredoxin changes and inflammatory cytokines support the involvement of neuro-inflammation and oxidative stress in Autism Spectrum Disorder. Journal of Translational Medicine. 2019;17.
7. Ahmad SF, Nadeem A, Ansari MA, Bakheet SA, Attia SM, Zoheir KMA, et al. Imbalance between the anti- and pro-inflammatory milieu in blood leukocytes of autistic children. Molecular Immunology. 2017;82:57-65.
8. Ahmad SF, Nadeem A, Ansari MA, Bakheet SA, Al-Ayadhi LY, Attia SM. Upregulation of IL-9 and JAK-STAT signaling pathway in children with autism. Progress in Neuro-Psychopharmacology and Biological Psychiatry. 2017;79:472-80.
9. Ahmad SF, Ansari MA, Ahmed N, Bakheet SA, Al-Ayadhi LY, Attia SM. Elevated IL-16 expression is associated with development of immune dysfunction in children with autism. Psychopharmacology. 2019;236(2):831-8.
10. Ahmad SF, Ansari MA, Nadeem A, Bakheet SA, Al-Ayadhi LY, Alotaibi MR, et al. Dysregulation of T cell immunoglobulin and mucin domain 3 (TIM-3) signaling in peripheral immune cells is associated with immune dysfunction in autistic children. Molecular Immunology. 2019;106:77-86.
11. Ahmad SF, Ansari MA, Nadeem A, Bakheet SA, Al-Ayadhi LY, Alasmari AF, et al. Involvement of CD45 cells in the development of autism spectrum disorder through dysregulation of granulocyte-macrophage colony-stimulating factor, key inflammatory cytokines, and transcription factors. Int Immunopharmacol. 2020;83:106466.
12. Akintunde ME, Rose M, Krakowiak P, Heuer L, Ashwood P, Hansen R, et al. Increased production of IL-17 in children with autism spectrum disorders and co-morbid asthma. J Neuroimmunol. 2015;286:33-41.
13. Al-Ayadhi LY. Pro-inflammatory cytokines in autistic children in central Saudi Arabia. Neurosciences (Riyadh). 2005;10(2):155-8.
14. Al-ayadhi LY, Mostafa GA. Increased serum osteopontin levels in autistic children: relation to the disease severity. Brain Behav Immun. 2011;25(7):1393-8.

15. Al-Ayadhi LY, Mostafa GA. Elevated serum levels of interleukin-17A in children with autism. Journal of Neuroinflammation. 2012;9.
16. Al-Ayadhi LY, Mostafa GA. Elevated serum levels of macrophage-derived chemokine and thymus and activation-regulated chemokine in autistic children. Journal of Neuroinflammation. 2013;10.
17. Alzghoul L, Abdelhamid SS, Yanis AH, Qwaider YZ, Aldahabi M, Albdour SA. The association between levels of inflammatory markers in autistic children compared to their unaffected siblings and unrelated healthy controls. Turkish Journal of Medical Sciences. 2019;49(4):1047-53.
18. Ashaat EA, Taman KH, Kholoussi N, El Ruby MO, Zaki ME, El Wakeel MA, et al. Altered adaptive cellular immune function in a group of Egyptian children with autism. Journal of Clinical and Diagnostic Research. 2017;11(10):SC14-SC7.
19. Ashwood P. Differential T cell levels of tumor necrosis factor receptor-II in children with autism. Frontiers in Psychiatry. 2018;9.
20. Ashwood P, Enstrom A, Krakowiak P, Hertz-Picciotto I, Hansen RL, Croen LA, et al. Decreased transforming growth factor beta1 in autism: A potential link between immune dysregulation and impairment in clinical behavioral outcomes. Journal of Neuroimmunology. 2008;204(1-2):149-53.
21. Ashwood P, Schauer J, Pessah IN, Van de Water J. Preliminary evidence of the in vitro effects of BDE-47 on innate immune responses in children with autism spectrum disorders. J Neuroimmunol. 2009;208(1-2):130-5.
22. Ashwood P, Corbett BA, Kantor A, Schulman H, van de Water J, Amaral DG. In search of cellular immunophenotypes in the blood of children with autism. PLoS ONE. 2011;6(5).
23. Ashwood P, Krakowiak P, Hertz-Picciotto I, Hansen R, Pessah I, Van de Water J. Elevated plasma cytokines in autism spectrum disorders provide evidence of immune dysfunction and are associated with impaired behavioral outcome. Brain, Behavior, and Immunity. 2011;25(1):40-5.
24. Ashwood P, Krakowiak P, Hertz-Picciotto I, Hansen R, Pessah IN, Van de Water J. Altered T cell responses in children with autism. Brain Behav Immun. 2011;25(5):840-9.
25. Ashwood P, Krakowiak P, Hertz-Picciotto I, Hansen R, Pessah IN, Van de Water J. Associations of impaired behaviors with elevated plasma chemokines in autism spectrum disorders. Journal of Neuroimmunology. 2011;232(1-2):196-9.
26. Atwan H, Assarehzadegan MA, Shekarabi M, Jazayeri SM, Barfi S, Shoormasti RS, et al. Assessment of miR-181b-5p, miR-23a-3p, BCL-2, and IL-6 in peripheral blood mononuclear cells of autistic patients; likelihood of reliable biomarkers. Iranian Journal of Allergy, Asthma and Immunology. 2020;19(1):74-83.
27. Balestrieri E, Cipriani C, Matteucci C, Benvenuto A, Coniglio A, Argaw-Denboba A, et al. Children with autism spectrum disorder and their mothers share abnormal expression of selected endogenous retroviruses families and cytokines. Frontiers in Immunology. 2019;10(SEP).
28. Barbosa IG, Rodrigues DH, Rocha NP, Sousa LFDC, Vieira ELM, Simões-e-Silva AC, et al. Plasma levels of alarmin IL-33 are unchanged in autism spectrum disorder: A preliminary study. Journal of Neuroimmunology. 2015;278:69-72.

29. Basheer S, Venkataswamy MM, Christopher R, Van Amelsvoort T, Srinath S, Girimaji SC, et al. Immune aberrations in children with Autism Spectrum Disorder: a case-control study from a tertiary care neuropsychiatric hospital in India. Psychoneuroendocrinology. 2018;94:162-7.
30. Bennabi M, Tarantino N, Gaman A, Scheid I, Krishnamoorthy R, Debré P, et al. Persistence of dysfunctional natural killer cells in adults with high-functioning autism spectrum disorders: stigma/consequence of unresolved early infectious events? Molecular Autism. 2019;10.
31. Bijl N, Thys C, Wittevrongel C, De la Marche W, Devriendt K, Peeters H, et al. Platelet studies in autism spectrum disorder patients and first-degree relatives. Molecular Autism. 2015;6.
32. Breece E, Paciotti B, Nordahl CW, Ozonoff S, Van de Water JA, Rogers SJ, et al. Myeloid dendritic cells frequencies are increased in children with autism spectrum disorder and associated with amygdala volume and repetitive behaviors. Brain, Behavior, and Immunity. 2013;31:69-75.
33. Bressler JP, Gillin PK, O'Driscoll C, Kiihl S, Solomon M, Zimmerman AW. Maternal antibody reactivity to lymphocytes of offspring with autism. Pediatric Neurology. 2012;47(5):337-40.
34. Bryn V, Aass HCD, Skjeldal OH, Isaksen J, Saugstad OD, Ormstad H. Cytokine Profile in Autism Spectrum Disorders in Children. Journal of Molecular Neuroscience. 2017;61(1).
35. Businaro R, Corsi M, Azzara G, Di Raimo T, Laviola G, Romano E, et al. Interleukin-18 modulation in autism spectrum disorders. J Neuroinflammation. 2016;13:2.
36. Carissimi C, Laudadio I, Palone F, Fulci V, Cesi V, Cardona F, et al. Functional analysis of gut microbiota and immunoinflammation in children with autism spectrum disorders. Digestive and Liver Disease. 2019;51(10):1366-74.
37. Chun-Chun H, Xu X, Guo-Liang X, Xu Q, Bing-Rui Z, Chun-Yang L, et al. Alterations in plasma cytokine levels in chinese children with autism spectrum disorder. Autism Research. 2018;11(7):989-99.
38. Croonenberghs J, Bosmans E, Deboutte D, Kenis G, Maes M. Activation of the inflammatory response system in autism. Neuropsychobiology. 2002;45(1):1-6.
39. Denney DR, Frei BW, Gaffney GR. Lymphocyte subsets and interleukin-2 receptors in autistic children. Journal of Autism and Developmental Disorders. 1996;26(1):87-97.
40. Eftekharian MM, Ghafouri-Fard S, Noroozi R, Omrani MD, Arsang-jang S, Ganji M, et al. Cytokine profile in autistic patients. Cytokine. 2018;108:120-6.
41. El Gohary TM, El Aziz NA, Darweesh M, Sadaa ES. Plasma level of transforming growth factor β 1 in children with autism spectrum disorder. Egyptian Journal of Ear, Nose, Throat and Allied Sciences. 2015;16(1):69-73.
42. El Wakkad ASED, Saleh MT. The proinflammatory cytokines in children with autism. Pakistan Journal of Biological Sciences. 2006;9(14):2593-9.
43. El-Ansary A, Al-Ayadhi L. Neuroinflammation in autism spectrum disorders. Journal of Neuroinflammation. 2012;9:265.

44. El-Ansary A, Al-Ayadhi L. GABAergic/glutamatergic imbalance relative to excessive neuroinflammation in autism spectrum disorders. Journal of Neuroinflammation. 2014;11.
45. El-Ansary AK, Ben Bacha AG, Al-Ayadhi LY. Proinflammatory and proapoptotic markers in relation to mono and di-cations in plasma of autistic patients from Saudi Arabia. Journal of Neuroinflammation. 2011;8.
46. El-Ansary A, Hassan WM, Qasem H, Das UN. Identification of Biomarkers of Impaired Sensory Profiles among Autistic Patients. PLoS One. 2016;11(11).
47. Emanuele E, Orsi P, Boso M, Broglia D, Brondino N, Barale F, et al. Low-grade endotoxemia in patients with severe autism. Neuroscience Letters. 2010;471(3):162-5.
48. Engstrom HA, Ohlson S, Stubbs EG, Maciulis A, Caldwell V, Odell JD, et al. Decreased Expression of CD95 (FAS/APO-1) on CD4+ T-lymphocytes from Participants with Autism. Journal of Developmental and Physical Disabilities. 2003;15(2):155-63.
49. Enstrom AM, Lit L, Onore CE, Gregg JP, Hansen RL, Pessah IN, et al. Altered gene expression and function of peripheral blood natural killer cells in children with autism. Brain, Behavior, and Immunity. 2009;23(1):124-33.
50. Enstrom AM, Onore CE, Van de Water JA, Ashwood P. Differential monocyte responses to TLR ligands in children with autism spectrum disorders. Brain, Behavior, and Immunity. 2010;24(1):64-71.
51. Enstrom A, Onore C, Hertz-Picciotto I, Hansen R, Croen L, Van De Water J, et al. Detection of IL-17 and IL-23 in plasma samples of children with autism. American Journal of Biochemistry and Biotechnology. 2008;4(2):114-20.
52. Esnafoglu E, Ayyıldız SN. Decreased levels of serum fibroblast growth factor-2 in children with autism spectrum disorder. Psychiatry Research. 2017;257:79-83.
53. Ferrante P, Saresella M, Guerini FR, Marzorati M, Musetti MC, Cazzullo AG. Significant association of HLA A2-DR11 with CD4 naive decrease in autistic children. Biomed Pharmacother. 2003;57(8):372-4.
54. Ghaffari MA, Mousavinejad E, Riahi F, Mousavinejad M, Afsharmanesh MR. Increased Serum Levels of Tumor Necrosis Factor-Alpha, Resistin, and Visfatin in the Children with Autism Spectrum Disorders: A Case-Control Study. Neurology Research International. 2016;2016.
55. Gomez-Fernandez A, de la Torre-Aguilar MJ, Gil-Campos M, Flores-Rojas K, Cruz-Rico MD, Martin-Borreguero P, et al. Children with autism spectrum disorder with regression exhibit a different profile in plasma cytokines and adhesion molecules compared to children without such regression. Frontiers in Pediatrics. 2018;6.
56. Guloksuz SA, Abali O, Aktas Cetin E, Bilgic Gazioglu S, Deniz G, Yildirim A, et al. Elevated plasma concentrations of S100 calcium-binding protein B and tumor necrosis factor alpha in children with autism spectrum disorders. Braz J Psychiatry. 2017;39(3):195-200.
57. Gupta S, Aggarwal S, Rashanravan B, Lee T. Th1- and Th2-like cytokines in CD4+ and CD8+ T cells in autism. J Neuroimmunol. 1998;85(1):106-9.
58. Hamed NO, Laila Al A, Osman MA, Elkhawad AO, Bjørklund G, Qasem H, et al. Determination of neuroinflammatory biomarkers in autistic and neurotypical Saudi children. Metabolic Brain Disease. 2019;34(4):1049-60.

59. Han YM, Cheung WK, Wong CK, Sze SL, Cheng TW, Yeung MK, et al. Distinct Cytokine and Chemokine Profiles in Autism Spectrum Disorders. Front Immunol. 2017;8:11.
60. Hashim H, Abdelrahman H, Mohammed D, Karam R. Association between plasma levels of transforming growth factor-β1, IL-23 and IL-17 and the severity of autism in Egyptian children. Research in Autism Spectrum Disorders. 2013;7(1):199-204.
61. Heuer LS, Rose M, Ashwood P, Van de Water J. Decreased levels of total immunoglobulin in children with autism are not a result of B cell dysfunction. Journal of Neuroimmunology. 2012;251(1-2):94-102.
62. Heuer LS, Croen LA, Jones KL, Yoshida CK, Hansen RL, Yolken R, et al. An Exploratory Examination of Neonatal Cytokines and Chemokines as Predictors of Autism Risk: The Early Markers for Autism Study. Biological Psychiatry. 2019;86(4):255-64.
63. Hollander E, DelGiudice-Asch G, Simon L, Schmeidler J, et al. B lymphocyte antigen D8/17 and repetitive behaviors in autism. The American Journal of Psychiatry. 1999;156(2):317-20.
64. Hunter LC, O'Hare A, Herron WJ, Fisher LA, Jones GE. Opioid peptides and dipeptidyl peptidase in autism. Developmental Medicine and Child Neurology. 2003;45(2):121-8.
65. Inga Jácome MC, Morales Chacòn LM, Vera Cuesta H, Maragoto Rizo C, Whilby Santiesteban M, Ramos Hernandez L, et al. Peripheral Inflammatory Markers Contributing to Comorbidities in Autism. Behav Sci (Basel). 2016;6(4).
66. Jyonouchi H, Sun S, Le H. Proinflammatory and regulatory cytokine production associated with innate and adaptive immune responses in children with autism spectrum disorders and developmental regression. Journal of Neuroimmunology. 2001;120(1-2):170-9.
67. Jyonouchi H, Sun S, Itokazu N. Innate immunity associated with inflammatory responses and cytokine production against common dietary proteins in patients with autism spectrum disorder. Neuropsychobiology. 2002;46(2):76-84.
68. Jyonouchi H, Geng L, Ruby A, Zimmerman-Bier B. Dysregulated Innate Immune Responses in Young Children with Autism Spectrum Disorders: Their Relationship to Gastrointestinal Symptoms and Dietary Intervention. Neuropsychobiology. 2005;51(2):77-85.
69. Jyonouchi H, Geng L, Ruby A, Reddy C, Zimmerman-Bier B. Evaluation of an association between gastrointestinal symptoms and cytokine production against common dietary proteins in children with autism spectrum disorders. Journal of Pediatrics. 2005;146(5):605-10.
70. Jyonouchi H, Geng L, Cushing-Ruby A, Quraishi H. Impact of innate immunity in a subset of children with autism spectrum disorders: A case control study. Journal of Neuroinflammation. 2008;5.
71. Jyonouchi H, Geng L, Streck DL, Toruner GA. Children with autism spectrum disorders (ASD) who exhibit chronic gastrointestinal (GI) symptoms and marked fluctuation of behavioral symptoms exhibit distinct innate immune abnormalities and transcriptional profiles of peripheral blood (PB) monocytes. Journal of Neuroimmunology. 2011;238(1-2):73-80.
72. Jyonouchi H, Geng L, Streck DL, Toruner GA. Immunological characterization and transcription profiling of peripheral blood (PB) monocytes in children with autism spectrum disorders (ASD) and specific polysaccharide antibody deficiency (SPAD): case study. Journal of Neuroinflammation. 2012;9:4.
73. Jyonouchi H, Geng L, Davidow AL. Cytokine profiles by peripheral blood monocytes are associated with changes in behavioral symptoms following immune insults in a subset of ASD subjects: an inflammatory subtype? Journal of Neuroinflammation. 2014;11.

74. Jyonouchi H, Geng L, Streck DL, Dermody JJ, Toruner GA. MicroRNA expression changes in association with changes in interleukin-1β/interleukin10 ratios produced by monocytes in autism spectrum disorders: their association with neuropsychiatric symptoms and comorbid conditions (observational study). Journal of Neuroinflammation. 2017;14.
75. Jyonouchi H, Geng L, Rose S, Bennuri SC, Frye RE. Variations in mitochondrial respiration differ in IL-1ß/IL-10 ratio based subgroups in autism spectrum disorders. Frontiers in Psychiatry. 2019;10(FEB).
76. Kordulewska NK, Kostyra E, Piskorz-Ogórek K, Moszyńska M, Cieślińska A, Fiedorowicz E, et al. Serum cytokine levels in children with spectrum autism disorder: Differences in pro- and anti-inflammatory balance. Journal of Neuroimmunology. 2019;337.
77. Krakowiak P, Goines PE, Tancredi DJ, Ashwood P, Hansen RL, Hertz-Picciotto I, et al. Neonatal Cytokine Profiles Associated With Autism Spectrum Disorder. Biological Psychiatry. 2017;81(5):442-51.
78. Kutlu A, Cevher Bınıcı N. Does increased neutrophil-lymphocyte ratio predict autism spectrum disorder? Anadolu Psikiyatri Dergisi. 2018;19(6):607-14.
79. Kutuk MO, Tufan E, Gokcen C, Kilicaslan F, Karadag M, Mutluer T, et al. Cytokine expression profiles in Autism spectrum disorder: A multi-center study from Turkey. Cytokine. 2020;133.
80. Lochman I, Švachová V, Mílková Pavlíková K, Medřická H, Novák V, Trilecová L, et al. Serum cytokine and growth factor levels in children with autism spectrum disorder. Medical Science Monitor. 2018;24:2639-46.
81. López-Cacho JM, Gallardo S, Posada M, Aguerri M, Calzada D, Mayayo T, et al. Characterization of immune cell phenotypes in adults with autism spectrum disorders. Journal of Investigative Medicine. 2016;64(7):1179-85.
82. Magid-Bernstein J, Mahajan K, Lincoln J, Ming X, Rohowsky-Kochan C. Case report: Cytokine and CD4+ T-cell profiles of monozygotic twins with autism and divergent comorbidities and drug treatment. Journal of Child Neurology. 2015;30(3):386-90.
83. Makinodan M, Iwata K, Ikawa D, Yamashita Y, Yamamuro K, Toritsuka M, et al. Tumor necrosis factor-alpha expression in peripheral blood mononuclear cells correlates with early childhood social interaction in autism spectrum disorder. Neurochemistry International. 2017;104:1-5.
84. Malik M, Sheikh AM, Wen G, Spivack W, Brown WT, Li X. Expression of inflammatory cytokines, Bcl2 and cathepsin D are altered in lymphoblasts of autistic subjects. Immunobiology. 2011;216(1-2):80-5.
85. Manzardo AM, Henkhaus R, Dhillon S, Butler MG. Plasma cytokine levels in children with autistic disorder and unrelated siblings. International Journal of Developmental Neuroscience. 2012;30(2):121-7.
86. Mills JL, Hediger ML, Molloy CA, Chrousos GP, Manning-Courtney P, Yu KF, et al. Elevated levels of growth-related hormones in autism and autism spectrum disorder. Clinical Endocrinology. 2007;67(2):230-7.
87. Mizejewski GJ, Lindau-Shepard B, Pass KA. Newborn screening for autism: in search of candidate biomarkers. Biomarkers in Medicine. 2013;7(2):247-60.
88. Moaaz M, Youssry S, Elfatatry A, El Rahman MA. Th17/Treg cells imbalance and their related cytokines (IL-17, IL-10 and TGF-β) in children with autism spectrum disorder. Journal of Neuroimmunology. 2019;337.

89. Molloy CA, Morrow AL, Meinzen-Derr J, Schleifer K, Dienger K, Manning-Courtney P, et al. Elevated cytokine levels in children with autism spectrum disorder. Journal of Neuroimmunology. 2006;172(1-2):198-205.
90. Mostafa GA, Al-Ayadhi LY. The possible link between elevated serum levels of epithelial cell-derived neutrophil- activating peptide-78 (ENA-78/CXCL5) and autoimmunity in autistic children. Behavioral and Brain Functions. 2015;11(1):1.
91. Mostafa GA, Al Shehab A, Fouad NR. Frequency of CD4+CD25high regulatory T cells in the peripheral blood of Egyptian children with autism. Journal of Child Neurology. 2010;25(3):328-35.
92. Nadeem A, Ahmad SF, Attia SM, Al-Ayadhi LY, Bakheet SA, Al-Harbi NO. Oxidative and inflammatory mediators are upregulated in neutrophils of autistic children: Role of IL-17A receptor signaling. Progress in Neuro-Psychopharmacology and Biological Psychiatry. 2019;90:204-11.
93. Nadeem A, Ahmad SF, Al-Ayadhi LY, Attia SM, Al-Harbi NO, Alzahrani KS, et al. Differential regulation of Nrf2 is linked to elevated inflammation and nitrative stress in monocytes of children with autism. Psychoneuroendocrinology. 2020;113.
94. Nadeem A, Ahmad SF, Al-Harbi NO, Alasmari AF, Al-Ayadhi LY, Alasmari F, et al. Upregulation of enzymatic antioxidants in CD4+ T cells of autistic children. Biochimie. 2020;171-172:205-12.
95. Nadeem A, Ahmad SF, Attia SM, Al-Ayadhi LY, Al-Harbi NO, Bakheet SA. Dysregulation in IL-6 receptors is associated with upregulated IL-17A related signaling in CD4+ T cells of children with autism. Progress in Neuro-Psychopharmacology and Biological Psychiatry. 2020;97.
96. Napolioni V, Ober-Reynolds B, Szelinger S, Corneveaux JJ, Pawlowski T, Ober-Reynolds S, et al. Plasma cytokine profiling in sibling pairs discordant for autism spectrum disorder. Journal of Neuroinflammation. 2013;10:38.
97. Nelson PG, Kuddo T, Song EY, Dambrosia JM, Kohler S, Satyanarayana G, et al. Selected neurotrophins, neuropeptides, and cytokines: developmental trajectory and concentrations in neonatal blood of children with autism or Down syndrome. International Journal of Developmental Neuroscience. 2006;24(1):73-80.
98. Ning J, Xu L, Shen CQ, Zhang YY, Zhao Q. Increased serum levels of macrophage migration inhibitory factor in autism spectrum disorders. NeuroToxicology. 2019;71:1-5.
99. Okada K, Hashimoto K, Iwata Y, Nakamura K, Tsujii M, Tsuchiya KJ, et al. Decreased serum levels of transforming growth factor-β1 in patients with autism. Progress in Neuro-Psychopharmacology and Biological Psychiatry. 2007;31(1):187-90.
100. Onore C, Enstrom A, Krakowiak P, Hertz-Picciotto I, Hansen R, Van de Water J, et al. Decreased cellular IL-23 but not IL-17 production in children with autism spectrum disorders. J Neuroimmunol. 2009;216(1-2):126-9.
101. Pardo CA, Farmer CA, Thurm A, Shebl FM, Ilieva J, Kalra S, et al. Serum and cerebrospinal fluid immune mediators in children with autistic disorder: A longitudinal study. Molecular Autism. 2017;8(1).
102. Pecorelli A, Cervellati F, Belmonte G, Montagner G, Waldon P, Hayek J, et al. Cytokines profile and peripheral blood mononuclear cells morphology in Rett and autistic patients. Cytokine. 2016;77:180-8.
103. Plioplys AV, Greaves A, Kazemi K, Silverman E. Lymphocyte function in autism and rett syndrome. Neuropsychobiology. 1994;29(1):12-6.

104. Ramsey JM, Guest PC, Broek JA, Glennon JC, Rommelse N, Franke B, et al. Identification of an age-dependent biomarker signature in children and adolescents with autism spectrum disorders. Molecular Autism. 2013;4(1).
105. Ricci S, Businaro R, Ippoliti F, Lo Vasco VR, Massoni F, Onofri E, et al. Altered cytokine and BDNF levels in autism spectrum disorder. Neurotoxicity Research. 2013;24(4):491-501.
106. Rodrigues DH, Rocha NP, Sousa LF, Barbosa IG, Kummer A, Teixeira AL. Changes in adipokine levels in autism spectrum disorders. Neuropsychobiology. 2014;69(1):6-10.
107. Rose D, Ashwood P. Rapid communication: Plasma interleukin-35 in children with Autism. Brain Sciences. 2019;9(7).
108. Rose DR, Yang H, Serena G, Sturgeon C, Ma B, Careaga M, et al. Differential immune responses and microbiota profiles in children with autism spectrum disorders and co-morbid gastrointestinal symptoms. Brain, Behavior, and Immunity. 2018;70:354-68.
109. Russo AJ. Correlation Between Hepatocyte Growth Factor (HGF) and Gamma-Aminobutyric Acid (GABA) Plasma Levels in Autistic Children. Biomark Insights. 2013;8:69-75.
110. Russo AJ. Increased Epidermal Growth Factor Receptor (EGFR) Associated with Hepatocyte Growth Factor (HGF) and Symptom Severity in Children with Autism Spectrum Disorders (ASDs). Journal of Central Nervous System Disease. 2014;6:JCNSD.S13767.
111. Russo AJ. Decreased Phosphorylated Protein Kinase B (Akt) in Individuals with Autism Associated with High Epidermal Growth Factor Receptor (EGFR) and Low Gamma-Aminobutyric Acid (GABA). Biomarker Insights. 2015;10:BMI.S21946.
112. Russo AJ, Krigsman A, Jepson B, Wakefield A. Decreased Serum Hepatocyte Growth Factor (HGF) in Autistic Children with severe Gastrointestinal Disease. Biomarker Insights. 2009;4:BMI.S3656.
113. Saad K, Zahran AM, Elsayh KI, Abdel-rahman AA, Al-atram AA, Hussein A, et al. Frequency of Dendritic Cells and Their Expression of Costimulatory Molecules in Children with Autism Spectrum Disorders. Journal of Autism and Developmental Disorders. 2017;47(9):2671-8.
114. Saad K, Abdallah AM, Abdel-Rahman AA, Al-Atram AA, Abdel-Raheem YF, Gad EF, et al. Polymorphism of interleukin-1β and interleukin-1 receptor antagonist genes in children with autism spectrum disorders. Prog Neuropsychopharmacol Biol Psychiatry. 2020;103:109999.
115. Saresella M, Marventano I, Guerini FR, Mancuso R, Ceresa L, Zanzottera M, et al. An Autistic Endophenotype Results in Complex Immune Dysfunction in Healthy Siblings of Autistic Children. Biological Psychiatry. 2009;66(10):978-84.
116. Saresella M, Piancone F, Marventano I, Zoppis M, Hernis A, Zanette M, et al. Multiple inflammasome complexes are activated in autistic spectrum disorders. Brain, Behavior, and Immunity. 2016;57:125-33.
117. Shen L, Feng C, Zhang K, Chen Y, Gao Y, Ke J, et al. Proteomics study of peripheral blood mononuclear cells (PBMCs) in autistic children. Frontiers in Cellular Neuroscience. 2019;13.
118. Shen Y, Oua J, Liu M, Shi L, Li Y, Xiao L, et al. Altered plasma levels of chemokines in autism and their association with social behaviors. Psychiatry Research. 2016;244:300-5.
119. Shen Y, Li Y, Shi L, Liu M, Wu R, Xia K, et al. Autism spectrum disorder and severe social impairment associated with elevated plasma interleukin-8. Pediatr Res. 2020.

120. Singh S, Yazdani U, Gadad B, Zaman S, Hynan LS, Roatch N, et al. Serum thyroid-stimulating hormone and interleukin-8 levels in boys with autism spectrum disorder. Journal of Neuroinflammation. 2017;14.
121. Singh VK. Plasma increase of interleukin-12 and interferon-gamma Pathological significance in autism. Journal of Neuroimmunology. 1996;66(1-2):143-5.
122. Singh VK, Fudenberg HH, Emerson D, Coleman M. Immunodiagnosis and immunotherapy in autistic children. Ann N Y Acad Sci. 1988;540:602-4.
123. Singh VK, Warren RP, Odell JD, Cole P. Changes of soluble interleukin-2, interleukin-2 receptor, T8 antigen, and interleukin-1 in the serum of autistic children. Clinical Immunology and Immunopathology. 1991;61(3):448-55.
124. Siniscalco D, Mijatovic T, Bosmans E, Cirillo A, Kruzliak P, Lombardi VC, et al. Decreased numbers of CD57+CD3-cells identify potential innate immune differences in patients with autism spectrum disorder. In Vivo. 2016;30(2):83-90.
125. Stubbs EG, Crawford ML. Depressed lymphocyte responsiveness in autistic children. J Autism Child Schizophr. 1977;7(1):49-55.
126. Suzuki K, Hashimoto K, Iwata Y, Nakamura K, Tsujii M, Tsuchiya KJ, et al. Decreased Serum Levels of Epidermal Growth Factor in Adult Subjects with High-Functioning Autism. Biological Psychiatry. 2007;62(3):267-9.
127. Suzuki K, Matsuzaki H, Iwata K, Kameno Y, Shimmura C, Kawai S, et al. Plasma Cytokine Profiles in Subjects with High-Functioning Autism Spectrum Disorders. PLoS One. 2011;6(5).
128. Sweeten TL, Posey DJ, McDougle CJ. High blood monocyte counts and neopterin levels in children with autistic disorder. The American Journal of Psychiatry. 2003;160(9):1691-3.
129. Sweeten TL, Posey DJ, Shankar S, McDougle CJ. High nitric oxide production in autistic disorder: A possible role for interferon-γ. Biological Psychiatry. 2004;55(4):434-7.
130. Tobiasova Z, van der Lingen KHB, Scahill L, Leckman JF, Zhang Y, Chae W, et al. Risperidone-Related Improvement of Irritability in Children with Autism Is not Associated with Changes in Serum of Epidermal Growth Factor and Interleukin-13. Journal of Child and Adolescent Psychopharmacology. 2011;21(6):555-64.
131. Tonhajzerova I, Ondrejka I, Mestanik M, Mikolka P, Hrtanek I, Mestanikova A, et al. Inflammatory Activity in Autism Spectrum Disorder. Adv Exp Med Biol. 2015;861:93-8.
132. Tostes MHFS, Teixeira HC, Gattaz WF, Brandão MAF, Raposo NRB. Altered neurotrophin, neuropeptide, cytokines and nitric oxide levels in autism. Pharmacopsychiatry. 2012;45(6):241-3.
133. Tsilioni I, Theoharides TC. Extracellular vesicles are increased in the serum of children with autism spectrum disorder, contain mitochondrial DNA, and stimulate human microglia to secrete IL-1β. Journal of Neuroinflammation. 2018;15.
134. Tsilioni I, Taliou A, Francis K, Theoharides TC. Children with autism spectrum disorders, who improved with a luteolin-containing dietary formulation, show reduced serum levels of TNF and IL-6. Translational Psychiatry. 2015;5(9).
135. Tural Hesapcioglu S, Kasak M, Cıtak Kurt AN, Ceylan MF. High monocyte level and low lymphocyte to monocyte ratio in autism spectrum disorders. International Journal of Developmental Disabilities. 2019;65(2):73-81.

136. Vojdani A, Mumper E, Granpeesheh D, Mielke L, Traver D, Bock K, et al. Low natural killer cell cytotoxic activity in autism: The role of glutathione, IL-2 and IL-15. Journal of Neuroimmunology. 2008;205(1-2):148-54.
137. Warren RP, Margaretten NC, Pace NC, Foster A. Immune abnormalities in patients with autism. Journal of Autism and Developmental Disorders. 1986;16(2):189-97.
138. Warren RP, Foster A, Margaretten NC. Reduced Natural Killer Cell Activity in Autism. Journal of the American Academy of Child and Adolescent Psychiatry. 1987;26(3):333-5.
139. Warren RP, Yonk LJ, Burger RA, Cole P, Odell JD, Warren WL, et al. Deficiency of suppressor-inducer (CD4+CD45RA+) T cells in autism. Immunol Invest. 1990;19(3):245-51.
140. Warren RP, Yonk J, Burger RW, Odell D, Warren WL. DR-positive T cells in autism: association with decreased plasma levels of the complement C4B protein. Neuropsychobiology. 1995;31(2):53-7.
141. Wasilewska J, Kaczmarski M, Stasiak-Barmuta A, Tobolczyk J, Kowalewska E. Low serum IgA and increased expression of CD23 on B lymphocytes in peripheral blood in children with regressive autism aged 3-6 years old. Archives of Medical Science. 2012;8(2):324-31.
142. Xie J, Huang L, Li X, Li H, Zhou Y, Zhu H, et al. Immunological cytokine profiling identifies TNF-α as a key molecule dysregulated in autistic children. Oncotarget. 2017;8(47):82390-8.
143. Yang CJ, Liu CL, Sang B, Zhu XM, Du YJ. The combined role of serotonin and interleukin-6 as biomarker for autism. Neuroscience. 2015;284:290-6.
144. Yonk LJ, Warren RP, Burger RA, Cole P, Odell JD, Warren WL, et al. CD4+ helper T cell depression in autism. Immunology Letters. 1990;25(4):341-5.
145. Zerbo O, Yoshida C, Grether JK, Van de Water J, Ashwood P, Delorenze GN, et al. Neonatal cytokines and chemokines and risk of Autism Spectrum Disorder: the Early Markers for Autism (EMA) study: a case-control study. Journal of Neuroinflammation. 2014;11:113.
146. Zimmerman AW, Jyonouchi H, Comi AM, Connors SL, Milstien S, Varsou A, et al. Cerebrospinal fluid and serum markers of inflammation in autism. Pediatric Neurology. 2005;33(3):195-201.
